# Reconceptualizing bipolar disorder through an integrative biological lens: synthesis, convergence, and emerging directions

**DOI:** 10.3389/fnint.2026.1775109

**Published:** 2026-07-07

**Authors:** Yasin Ali Muhammad

**Affiliations:** Department of Biology, Georgia State University, Atlanta, GA, United States

**Keywords:** bipolar disorder, chronic disease, integrative model, mitochondrial dysfunction, mood disorders, systems biology

## Abstract

Bipolar disorder (BD) is a highly complex, chronic psychiatric illness marked by episodic disturbances of mood. While historically conceptualized as a disorder of neurotransmitter imbalance, converging evidence suggests it is better understood as a disorder of dynamic dysregulation across multiple interacting systems, including genetic and epigenetic vulnerability, HPA axis dysregulation, mitochondrial/metabolic dysfunction, immune activation, circadian disruption, and gut-brain axis disturbances. These systems comprise a hierarchically organized, bidirectionally coupled feedback network that is responsible for the oscillatory mood states that define the disorder. Here, it’s proposed that a specific, testable, integrative model in which genetic/epigenetic vulnerabilities lead to destabilization of HPA axis regulation, which then leads to mitochondrial bioenergetic dysfunction (the central integrative node of the cascade), which then, through its interaction with immune activation and gut-brain signaling, causes the excitation-inhibition imbalance underlying the clinical presentation of mood-state transitions. From this model, three empirically tractable predictions are made: that co-occurring inflammatory/metabolic/circadian dysregulation will constitute a biological subtype with earlier onset, more rapid cycling, and a more negative response to lithium; that neuroendocrine/metabolic disturbances will prospectively precede rather than simply co-occur with mood-state transitions; and that interventions targeting the metabolic-inflammatory interface will produce mood-stabilizing effects whose magnitude correlates with baseline biological severity rather than symptom severity. Throughout, the importance of state-dependence, trait-level features, and medication confounds is discussed. Finally, translational implications for metabolic stratification, circadian-focused intervention, and gut-brain modulation are outlined.

## Introduction

Bipolar disorder (BD), is a chronic psychiatric condition defined by recurrent episodes of mania (or hypomania) and depression (Oliva et al., 2024). These mood states differ considerably in their behavioral and cognitive profiles. Manic episodes typically involve elevated or irritable mood, reduced sleep need, rapid speech, impulsivity, and heightened goal-directed activity, while depressive episodes bring anhedonia, psychomotor slowing, cognitive difficulties, and in severe cases, suicidal ideation (Zhang et al., 2023). BD affects roughly 1%–2.4% of adults globally (Geoffroy et al., 2013; Zhang et al., 2023), with onset most often occurring in late adolescence or early adulthood. Beyond episodic mood disturbance, BD imposes a substantial functional and societal burden. Recurrent mood instability disrupts interpersonal relationships, employment, and financial stability, while significantly raising suicide risk. Even with current pharmacological and psychotherapeutic treatments, sustained remission is achieved in only a minority of patients (Renes et al., 2014). The disorder is further complicated by high rates of comorbidity - including anxiety disorders, substance use disorders, metabolic syndrome, and cardiovascular disease - pointing toward systemic biological dysregulation rather than a condition limited to the central nervous system (Bessonova et al., 2020; Wang et al., 2022a,b; Sinha et al., 2018; Yamagata et al., 2017).

BD has long been framed in terms of neurotransmitter imbalance, but that notion is an over-simplification. Evidence has implicated multiple interacting domains: genetic and epigenetic susceptibility, circadian rhythm disruption, mitochondrial and metabolic dysfunction, immune activation, and gut-brain signaling (Hyndych et al., 2025; Leboyer et al., 2012). Each of these domains has been studied extensively, though typically in isolation. As a result, the field lacks a coherent framework explaining how these systems interact to produce mood instability, heterogeneity in clinical presentation, and progression over time. This review departs from prior inquiries by proposing a specific, testable integrative model rather than a parallel account of independent biological domains. The central argument is that BD emerges from a cascade in which genetic and epigenetic vulnerabilities destabilize HPA axis regulation, which in turn impairs mitochondrial bioenergetic function, and that this metabolic insufficiency - interacting bidirectionally with immune activation and gut-brain signaling - produces the oscillatory excitation-inhibition imbalance expressed clinically as mood-state transitions. This cascade is not proposed as a fixed linear sequence but as a dynamic feedback system: metabolic stress amplifies inflammatory signaling, inflammatory signaling further undermines mitochondrial efficiency, and both loop back onto neuroendocrine regulation. Several testable predictions follow from this model. First, individuals carrying high-risk variants in FKBP5, GSK3β, and circadian clock genes should show earlier and more severe HPA dysregulation, and this neuroendocrine profile should mediate - rather than merely correlate with - downstream metabolic and inflammatory abnormalities. Second, co-occurring elevated inflammatory markers, reduced oxidative phosphorylation (OXPHOS) capacity, and circadian instability should identify a biological subtype characterized by earlier onset, faster cycling, and poorer response to lithium monotherapy. Third, interventions targeting the metabolic-inflammatory-axis - such as ketogenic dietary strategies or adjunctive anti-inflammatory supplementation - should produce mood-stabilizing effects that exceed those predicted by symptom-level models alone. These predictions are empirically tractable and provide a basis for the longitudinal, multimodal study designs this review is intended to engender. Notably, the biological mechanisms underlying mood-state transitions - particularly switches between depressive and manic states - remain poorly defined. Proposed biomarkers, including inflammatory cytokines, glutamatergic measures, and metabolic indicators, often lack clear state specificity and are frequently confounded by medication exposure. Widely used composite measures - such as Glx as a proxy for glutamatergic activity or cumulative cortisol indices - can obscure meaningful biological distinctions and limit interpretability. Together, these issues have blocked the development of mechanistic models capable of integrating findings across domains into a unified understanding of BD.

In response to these limitations, this review adopts a framework that treats such interactions as an organizing principle. A systems-level model here means integrating molecular and epigenetic information, cellular metabolism and mitochondrial function, endocrine and immune physiology, and neural circuit-level dynamics into a unified structure that tracks how disturbances propagate across these various systems. To facilitate this process, several specific aims guide this synthesis. First, distinctions are drawn between state-dependent findings, trait-like biological features, and medication-related effects. Second, commonly conflated biological signals - such as glutamate versus glutamine dynamics, state-specific versus cumulative cortisol measures, and mitochondrial versus systemic metabolic activity - are explicitly disentangled to improve mechanistic clarity. Third, an integrative model is proposed in which genetic vulnerability, environmental exposures, and physiological dysregulation converge to drive dynamic transitions between mood states. Three predictions follow that are directly testable in longitudinal multimodal studies: first, that co-occurring dysregulation across the inflammatory, metabolic, and circadian nodes will identify a biological subtype characterized by earlier onset, faster cycling, and poorer lithium response; second, that neuroendocrine and metabolic disturbances will precede rather than merely accompany mood-state transitions when tracked prospectively; and third, that interventions targeting the metabolic-inflammatory interface will produce mood-stabilizing effects whose magnitude correlates with baseline severity of mitochondrial and inflammatory markers rather than with symptom severity alone. Thus, BD is framed here as a disorder of interacting regulatory systems. Genetic and epigenetic factors establish baseline vulnerability in stress responsivity, neurotransmitter regulation, and circadian timing. Environmental stressors and circadian disruption perturb hypothalamic-pituitary-adrenal (HPA) axis function and sleep-wake rhythms, which in turn influence mitochondrial function and cellular energy metabolism. These metabolic changes interact with immune signaling and gut-derived inflammatory signals, collectively altering differential neuronal excitability. The consequences of these interactions result in oscillations in patients relative stability, expressed clinically as transitions between depressive, manic, and mixed states.

This review is intended for both researchers and clinically oriented audiences. For researchers, it highlights methodological limitations and identifies priorities for multimodal and longitudinal study design. For clinicians, it outlines how systems-level insights may inform emerging strategies such as metabolic and/or circadian-based interventions, and approaches targeting inflammation and gut-brain signaling. By drawing together findings across traditionally siloed domains and emphasizing mechanistic convergence, this review aims to provide a clearer framework for understanding BD and to support the development of more precise, biologically informed approaches to treatment and investigation.

## Methods

The current narrative review summarizes evidence linking neurobiological, metabolic, immunological, genetic, and gut-brain axis processes to BD pathophysiology. Articles were identified using PubMed, Scopus, and Google Scholar databases searching key terms until July 2025. Keywords were paired using Boolean operators: (“bipolar disorder”) AND (“mitochondrial dysfunction” OR “glutamate metabolism” OR “circadian rhythm genes” OR “COMT” OR “MTHFR” OR “FKBP5” OR “immune dysregulation” OR “inflammation” OR “gut-brain axis” OR “intestinal permeability” OR “microbiome”). Only articles published in English were included. While recent articles within the past 15 years were prioritized to best understand the current scientific viewpoint and evolving hypotheses, additional articles outside of this window were included if they were foundational to the topic (i.e., heritability twin studies or initial molecular discoveries). Human studies and animal research that explored proposed mechanisms, pathological differences between mood states, and translational implications were included. Articles were then manually screened by topic to include studies relevant to biological mechanisms involved in BD. For included articles, he quality of study design, relevance to understand mechanisms involved in BD, novelty, and translatability were assessed. Since several topics (i.e., metabolic abnormalities, leaky gut syndrome, gene-by-environment interactions) have been historically understudied in BD but are being increasingly reviewed by authors as biologically similar or mechanistically relevant to BD pathophysiology, these topics were also highlighted. Also included were emerging and overlapping themes (i.e., neuroinflammation, glutamine/glutamate dysregulation, circadian rhythm gene mutations, microbiome metabolites) to better identify potential overlap between systems. Therefore, the current review does not follow a systematic review or meta-analysis format. An interpretative narrative style was employed. Topics that are well-established, controversial, and lacking but mechanistically plausible are discussed.

### Diagnostic criteria and classification

The Diagnostic and Statistical Manual of Mental Disorders, Fifth Edition, Text Revision (DSM-5-TR; Florida Behavioral Health Center, 2021; Jain and Mitra, 2023) - the primary reference for the classification of mental health disorders - categorizes BD into four main types, maintaining the core diagnostic criteria and duration thresholds established in DSM-5:

- Bipolar I disorder: At least one manic episode is required, which may be preceded or followed by hypomanic or major depressive episodes.
- Bipolar II disorder: Requires at least one hypomanic episode and one major depressive episode, with no history of a full manic episode.
- Cyclothymic disorder: Involves at least 2 years (1 year for youth) of numerous periods of hypomanic and depressive symptoms that do not meet full criteria for hypomanic or major depressive episodes.
- Other specified/unspecified bipolar and related disorders: Includes clinically significant mood disturbances with bipolar features that do not meet the above criteria.

A manic episode is characterized by a distinct period of at least 1 week (or any duration if hospitalized) of abnormally and persistently elevated, expansive, or irritable mood and abnormally and persistently increased activity or energy, as manifested by at least three of the following symptoms (four if the mood is only irritable): inflated self-esteem or grandiosity, decreased need for sleep, more talkative than usual or pressure to keep talking, flight of ideas or racing thoughts, distractibility, increased goal-directed activity or psychomotor agitation, and excessive involvement in activities that have a high potential for painful consequences (Marzani and Price Neff, 2021). The mood disturbance is severe enough to cause impairment in social and occupational functioning or necessitate hospitalization to prevent harm to self or others, or there are psychotic features, and the episode is not attributable to the physiological effects of a substance or another medical condition. Hypomanic episodes are similar in symptoms but last for a shorter duration (≥4 consecutive days) and do not cause marked impairment in functioning or include psychosis. Major depressive episodes are defined as at least 2 weeks of depressed mood or loss of interest or pleasure, accompanied by at least five additional symptoms (e.g., sleep disturbance, psychomotor changes, fatigue, feelings of worthlessness or excessive guilt, impaired concentration, and suicidal ideation), causing clinically significant distress or impairment and not being attributable to substances or medical conditions (Marzani and Price Neff, 2021). The DSM-5-TR presents the formal diagnostic criteria for BD and includes separate criteria for Bipolar I Disorder, Bipolar II Disorder, cyclothymic disorder, and other specified or unspecified bipolar and related disorders. It retains the basic diagnostic structure and approach of the DSM-5, with distinctions between Bipolar I and II, cyclothymic disorder, and other specified/unspecified bipolar-related conditions. The DSM-5-TR incorporates several enhancements and clarifications that, while not altering the fundamental thresholds for BD diagnoses, contribute to improved diagnostic precision. Notably, there are minor updates to the text descriptions for criteria, a consolidation of specifiers such as “with mixed features,” “with anxious distress,” and severity specifiers, and additional guidance on substance- or medication-induced mood disturbances. The clinical foundations and boundaries of BD are established in this section, setting the stage for the subsequent sections that will delve into its biological and genetic underpinnings.

## Heritability and genetic risk of bipolar disorder

Heritability is the proportion of observed phenotypic variance that can be explained by genetic factors (Barry et al., 2023). Twin and family studies of BD have generally produced heritability estimates in the region of 60-80% (Barnett and Smoller, 2009; O’Connell and Coombes, 2021). A basic measure of heritability can be calculated using Falconer’s equation:


h⁢2=2⁢(r⁢M⁢Z-r⁢D⁢Z),

where rMZ and rDZ are the rates of concordance in MZ and DZ twins, respectively. Concordance is the percentage of twin pairs for which both individuals are affected by the disorder (Hallmayer et al., 2011). Twin designs are predicated on the assumption that MZ and DZ pairs experience equivalent quality of rearing environment or a range of equivalent environments. Importantly, heritability is not a property of a disorder but a population-specific estimate dependent on the environmental conditions from which it is derived—and a measure of variance in susceptibility across a population, not within individuals. Although heritability estimates for BD are typically derived from twin samples, they are understood to reflect genetic contributions to variance in the broader general population.

A source of confusion regarding heritability stems from the “missing heritability” phenomenon (Matthews and Turkheimer, 2022). Genome-wide association studies have implicated a number of risk loci for BD, but collectively these account for only 20–40% of the total heritability estimate (Johansson et al., 2019), the remainder of which is assumed to be explained by rare variants, structural variants, gene-gene interactions, and epigenetic mechanisms. Falconer’s equation does not directly measure how genes cause BD - it observes that concordance increases as genetic similarity increases, from 20% in DZ twins to 60% in MZ twins, and scales that relationship into a population-level estimate. Heritability is therefore better understood as an estimate of the effect of increasing genetic similarity on concordance, rather than a direct measure of genetic causation. This distinction is consequential: heritability is frequently interpreted as the proportion of BD caused by genes, when it is more precisely a scaled estimate of how concordance changes as genetic similarity increases, under the assumption that environmental conditions are held constant.

Family studies provide a complementary source of genetic attribution data. First-degree relatives of affected individuals are at significantly increased risk relative to the general population (Merikangas and Yu, 2002; Song et al., 2015), with Song et al. (2015) reporting a relative risk (RR) of 6.7 (95% CI 6.3–7.0). However, the absolute risk (AR) in this group was 3.1%, compared with 0.5% in matched controls (absolute risk difference: 2.6%). Table 1 summarizes these estimates, presenting the AR, absolute risk difference (ARD), and RR for first-degree relatives compared with matched population controls. Familial aggregation is therefore clearly present, but even among individuals at high familial risk the disorder is not an inevitable outcome - highlighting the role of environmental modifiers and gene-environment interactions:


AR=(Numberofcasesinthegroup)÷

(Total⁢number⁢in⁢the⁢group)

- Total relatives of BD probands (first-degree relatives): 2,119
- Total matched controls: 3,269
- Risk in first-degree relatives: 3.1% (0.031)
- Risk in matched controls: 0.5% (0.005)

**TABLE 1:** Absolute risk of developing bipolar disorder among first-degree relatives.

Category	AR in probands	AR in controls	ARD
Total first-degree relatives	3.1%	0.5%	2.6%
Father	2.4%	0.4%	2.0%
Male offspring–father	2.7%	0.4%	2.3%
Female offspring–father	2.2%	0.4%	1.8%
Mother	3.9%	0.6%	3.3%
Male offspring–mother	3.9%	0.6%	3.3%
Female offspring-mother	3.9%	0.6%	3.3%

This table presents the absolute risk (AR) of developing BD for different first-degree relatives of BD probands compared with matched population controls, along with the absolute risk difference (ARD) and relative risk (RR) estimates. AR represents the proportion of individuals in each proband subgroup who developed BD. ARD indicates the increase in risk attributable to being a first-degree relative of a BD proband. RR is the unadjusted relative risk comparing probands with controls, and 95% confidence intervals (CIs) are provided to indicate the precision of these estimates. Additional methodological details concerning the calculation of relative risks and confidence intervals are provided in this table of Song et al. (2015).

Absolute risk difference: Tells you how much the risk actually increases in the exposed group (first-degree relatives), which is a real-world probability, not just a ratio.


A⁢R⁢D=R⁢i⁢s⁢k⁢p⁢r⁢o⁢b⁢a⁢n⁢d⁢s-R⁢i⁢s⁢k⁢c⁢o⁢n⁢t⁢r⁢o⁢l=0.031-

0.005=0.026⁢(2.6%)

RR:


R⁢R=R⁢i⁢s⁢k⁢p⁢r⁢o⁢b⁢a⁢n⁢d⁢s/R⁢i⁢s⁢k⁢C⁢o⁢n⁢t⁢r⁢o⁢l⁢s=0.031/0.005

   =6.2(unadjusted).

Building on evidence from twin and family studies, large population-based investigations continue to affirm the substantial heritability of BD. In the largest family study to date, Kendler et al. (2020) analyzed data from more than two million individuals in the Swedish cohort and found that BD transmission was statistically homogeneous across family types-including intact, father-absent, and adoptive families-indicating that genetic liability persists even when environmental conditions vary. Although absolute risk remains relatively low, BD ranks among the most heritable psychiatric conditions. Parker et al. (2018) reported that 36.8% of BD patients had a family member with the disorder, with a slightly higher prevalence in bipolar I (41.2%) than in bipolar II (36.3%) and substantially higher than in families of unipolar depressed patients (18.5%).

Significant disparities in the diagnosis and burden of BD are evident across geographic regions and ethno-racial groups (Akinhanmi et al., 2018). Using Global Burden of Disease (GBD) data from 1990 to 2021, Jiang et al. (2025) examined trends across 204 countries and 21 territories and identified several key patterns. Economically developed regions generally face fewer environmental stressors and possess greater mental health resources, but these supports are disproportionately concentrated among socioeconomically advantaged “core” populations. Marginalized groups, despite representing a large share of the population, encounter higher life pressures, reduced access to care, and consequently greater psychiatric morbidity (Kirkbride et al., 2024). In contrast, developing regions experienced the steepest increases in incidence and prevalence over the past three decades, largely driven by adverse exposures such as bullying, childhood sexual abuse, and intimate partner violence.

Age-related trends also shape the global burden of BD. Adolescents aged 15–19 represent the most affected population, with prevalence and disability-adjusted life years (DALYs) increasing sharply among individuals aged 10–39 between 1990 and 2021, while rates in adults over 40 remained stable or declined. The authors attribute these shifts to heightened academic pressures, lifestyle changes linked to digital technology use, and circadian disruption from excessive screen exposure, which collectively impair prefrontal cortex development and mood regulation. By contrast, middle-aged and older adults often benefit from more established coping mechanisms and social support, partially mitigating illness burden. However, under-recognized adolescent-onset BD may persist into adulthood, amplifying long-term psychosocial and medical consequences.

Sex differences also contribute to the burden of disease. Women have higher rates of disability due to BD, perhaps in response to fluctuations of hormones during puberty, pregnancy, postpartum, and menopause. Changes in luteinizing hormone and estradiol are associated with differences in stress reactivity that impact mood lability (Zhang and Swaab, 2024). Misdiagnosis can also be greater for individuals of African ancestry compared to other ethnic groups, with increased risk of being misdiagnosed with schizophrenia or unipolar depression (Akinhanmi et al., 2018). Bipolar depression that is misdiagnosed as unipolar depression may result in antidepressant-induced mania, while misdiagnosing BD as schizophrenia may preclude the use of lithium and mood stabilizers. Reducing diagnostic error rates with structured diagnostic interviews and blinded multi-racial consensus panels significantly improves diagnosis (Strakowski et al., 1996, 2003). GWAS have identified multiple risk loci, each of which has a small effect size (Cross-Disorder Group of the Psychiatric Genomics Consortium et al., 2013). The polygenic nature of BD suggests that many such variants with small effect sizes act together to confer risk. This is also reflected by the “missing heritability” in which heritability estimates using common variants only account for 20%–40% of total genetic variation, a much smaller proportion than the 60%–80% that is estimated in twin studies (Johansson et al., 2019). The “missing heritability” could be due to a number of other factors including rare variants, gene–gene or gene–environment interactions, or epigenetic effects, such as methylation of DNA and modification of histones (Johansson et al., 2019).

In summary, BD is among the most highly heritable of psychiatric disorders, with converging lines of evidence from family studies, twin studies, and population-based genetic research. First-degree relatives are at a greater relative risk, but overall absolute risk is low, reflecting that there is vulnerability to disease, but not predestination. Geographic, socioeconomic, ethno-racial differences, as well as sex and age-related differences in hormones and development contribute to differences in disease burden and risk. Finally, GWAS have established that BD is a highly polygenic disorder that is influenced by the environment, which will be further explored through the lens of epigenetics and gene-environment interactions below.

## The hypothalamic-pituitary-adrenal-axis: molecular genetics stress, trauma, and activity

### Molecular genetics and epigenetic mechanisms concerning BD

Epigenetic mechanisms underpin a key mechanistic pathway by which stress influences biological susceptibility to BD. These mechanisms, which include DNA methylation, histone modification, chromatin remodeling, and noncoding RNA, act to control gene activity and expression, rather than change gene sequence. They dynamically modulate gene activity in response to stressors and developmental timing. Epigenetic processes are increasingly implicated in the pathophysiology of BD. There is growing evidence that BD is influenced by the epigenetic dysregulation of stress-related genes due to early-life adversity and illness stage (Hesam-Shariati et al., 2022; Legrand et al., 2021; Ludwig and Dwivedi, 2016; Fries et al., 2016). Childhood trauma has been shown to be associated with long-lasting methylation changes in genes related to stress responsivity, and these epigenetic alterations can have downstream effects on neuroendocrine function and mood regulation that persist into adulthood (Weaver, 2009; Mitchell et al., 2016; Roth et al., 2009).

FKBP5, a gene encoding the FK506-binding protein 51 (FKBP51), is one of the most consistently implicated epigenetic targets. FKBP51 is a co-chaperone that modulates glucocorticoid receptor (GR) sensitivity and influences stress response (Criado-Marrero et al., 2020). The methylation status of intronic regulatory regions in the FKBP5 gene plays a crucial role in controlling the gene’s transcriptional activity. In early-stage BD, higher methylation levels in the FKBP5 intronic regions are associated with lower FKBP51 expression. This maintains GR sensitivity and supports a functional negative feedback loop in the HPA axis (Fries et al., 2017). In contrast, chronic illness duration and cumulative stress exposure are associated with cortisol-induced hypomethylation of FKBP5, leading to overexpression of FKBP51. This dampens GR signaling and impairs the termination of the stress response (Criado-Marrero et al., 2020). This epigenetic shift, therefore, represents a transition from a state of adaptive stress regulation to a maladaptive endocrine phenotype and parallels the progression from early to late-stage BD (Fries et al., 2014).

This dysregulation is also physiologically reflected in a blunted response to the dexamethasone suppression test (DST). Reduced cortisol suppression on DST in BD patients is indicative of GR resistance and sustained HPA axis activation. Reduced FKBP5 methylation has also been found in other groups with prolonged psychosocial stress exposure, such as in female caregivers with blunted DST responses, suggesting a loss of GR sensitivity through a shared epigenetic mechanism (Palma-Gudiel et al., 2021). During manic episodes, heightened arousal and cortisol may further disrupt methylation homeostasis, amplifying FKBP51 expression and further weakening GR-mediated negative feedback (Saito et al., 2020). Mechanistically, excess FKBP51 competes with cortisol for binding to GR and disrupts the transport of the cortisol-GR complex into the nucleus, thereby impeding effective autoregulation of cortisol production and perpetuating HPA axis hyperactivity (Binder, 2009).

Prolonged FKBP5 hypomethylation in BD is associated with chronic HPA axis activation and decreased stress resilience (Großmann et al., 2024). This endocrine profile may contribute to increased vulnerability to mood episode recurrence and is also associated with cognitive decline observed in late-stage BD (Malekpour et al., 2023). Cortisol excess is neurotoxic to hippocampal neurons, impairs memory consolidation, and promotes neurodegenerative risk, providing a possible mechanistic link between mood instability and long-term cognitive outcomes in BD (Ouanes and Popp, 2019). Thus, epigenetically mediated GR resistance may not only contribute to mood dysregulation, but also to age-related cognitive vulnerability.

Taken together, epigenetic changes, particularly DNA methylation, act as a molecular pathway through which environmental adversity and genetic predisposition are integrated in BD. Dynamic methylation changes at stress-related genes, such as FKBP5, recalibrate glucocorticoid signaling and progressively weaken HPA axis feedback over time, decreasing resilience to stressors. Framed in this way, epigenetic dysregulation is not merely associative, but etiologically relevant and on a trajectory of biological remodeling that links early adversity to neuroendocrine instability, mood lability, and cognitive load. This epigenetic substrate provides a mechanistic bridge to the following discussion on functional genetic variants, such as COMT and MTHFR polymorphisms, that also shape neurotransmitter metabolism and stress reactivity in BD.

The enzyme catechol-O-methyltransferase (COMT) is a key regulator of catecholamine turnover, including dopamine, norepinephrine, and epinephrine (Antypa et al., 2013). COMT-catalyzed methylation reactions require S-adenosyl-L-methionine (SAMe) as a methyl donor and, consequently, reduced SAMe availability or accumulation of its byproduct, S-adenosylhomocysteine (SAH), act to constrain COMT activity (Zhu, 2002). Given that cortical dopamine tone critically influences affective stability and impulse control, functional variation in COMT has been investigated as a putative molecular contributor to BD (Wang et al., 2015).

Val158Met (rs4680) is the most well-studied functional polymorphism in COMT. A methionine (Met) substitution for valine (Val) at codon 158 alters the thermostability and enzymatic efficiency of COMT, and the Val allele results in three- to four-fold greater COMT activity relative to the Met allele (Antypa et al., 2013). Val carriers thus show increased dopamine degradation, whereas Met carriers have slower dopamine clearance and higher baseline dopamine levels, particularly in the prefrontal cortex (Yavich et al., 2007). The regional specificity of this effect is notable because dopamine transporter density in the prefrontal cortex is relatively low; COMT is therefore responsible for a substantial majority of cortical dopamine inactivation (>60%) (Liu et al., 2010; Yavich et al., 2007). Thus, functional genetic variation at COMT Val158Met directly modulates prefrontal dopaminergic tone.

The role of this regulatory axis in emotional regulation is further supported by preclinical studies. In rodents, COMT knockdown or inhibition upregulates prefrontal dopamine signaling and emotional reactivity. In parallel, it produces impairments in cognitive domains that show high co-variation with mood lability in BD (Burdick et al., 2007). Experimental elevation of dopamine produces mania-like phenotypes in preclinical models - including increased goal-directed behavior, reduced need for sleep, and impulsivity - supporting dopaminergic overdrive as a contributor to mood-state dysregulation (Siddique et al., 2018). Impaired termination of dopaminergic signaling is more broadly implicated in disorders with disinhibition and affective lability, including BD, schizophrenia, and addiction (Speranza et al., 2021). In some cohorts, the low-activity Met/Met genotype has been associated with greater affective instability and increased BD risk (Taylor, 2018), consistent with the hypothesis that reduced efficiency of cortical dopamine clearance may lower the threshold for a manic or hypomanic state.

Catechol-O-methyltransferase function is not genetically isolated but embedded within the broader methylation environment. MTHFR, a key enzyme in one-carbon metabolism, regulates the cycling of folate and the availability of methyl groups required for SAMe regeneration. Two common MTHFR polymorphisms, C677T and A1298C, have been found to reduce MTHFR activity and may result in a limited supply of methyl donors (Wang et al., 2015). Reduced methyl availability can also further limit COMT activity by both decreasing SAMe and increasing SAH, thus amplifying the functional consequences of low-activity COMT variants (Gao et al., 2018). This gene-gene interaction has potential clinical relevance in BD. When intrinsic COMT activity is lowered (Met allele) and methylation capacity is simultaneously impacted by MTHFR variation, cortical dopamine clearance may become disproportionately inefficient. Elevated dopamine tone may then bias mesocorticolimbic signaling toward increased D3 receptor engagement (Holly et al., 2015), a receptor implicated in rewards sensitivity, mood regulation, and cognitive flexibility. Within an “inverted U” model of prefrontal dopamine function, both insufficient and excessive dopamine negatively impact executive control and emotional regulation. People with combined COMT and MTHFR vulnerability may be predisposed to reside on the hyperdopaminergic limb of this curve, increasing their susceptibility to manic activation and affective volatility.

### Circadian rhythm-related genetics

Sleep-wake disruption is among the most robust clinical features of BD and is baked into diagnostic criteria. Disturbances of insomnia and hypersomnia both map onto mood state in patterned ways. Steinan et al. (2016) found in a BD cohort that hypersomnia was most strongly associated with depressive and mixed states, particularly in young patients and in those with bipolar I, but it was not clearly associated with “pure” (hypo)mania. Insomnia, by contrast, was very common in bipolar depression (34% in bipolar I and 69.7% in bipolar II) and was also prevalent in (hypo)manic bipolar I patients (20.6%), indicating that reduced sleep is not only a manic feature but also a bipolar depression marker. Beyond episodes, sleep fragmentation, increased sleep onset latency, higher wake after sleep onset, and irregularity in sleep timing are also reliably documented even during interepisode periods. Familial- and clinical high-risk individuals for BD already have reduced rest-activity cycle amplitude and higher night-to-night sleep efficiency variability (Ng et al., 2015). Longitudinal work using wearable devices has shown that circadian phase shifts and unstable sleep-wake rhythms often precede mood changes, particularly depressive worsening (Lim et al., 2024; Song et al., 2024). At the same time, BD populations are enriched for an evening chronotype, which is associated with earlier age at onset, rapid cycling, and lower nocturnal melatonin secretion, especially in bipolar I (Nurnberger et al., 2000; Zou H. et al., 2022; Zou P. et al., 2022). One could then argue that sleep and circadian instability are not just symptoms of episodes; they are early markers of mood destabilization and the illness biology itself.

Behavioral and physiological instability map onto variation in the genes that control the circadian clock. The circadian system itself is maintained by an intracellular transcription-translation feedback loop centered on CLOCK and BMAL1 (also known as ARNTL), which drive rhythmic expression of downstream clock-controlled genes, and PER and CRY proteins, which feedback to inhibit that drive. Additional regulators such as glycogen synthase kinase 3β (GSK3β) fine-tune timing within the suprachiasmatic nucleus and other brain regions that govern arousal, rewards, and mood. Disruption in components of the molecular clock affects mood-related behavior in animal models and appears to shape age of onset, episode recurrence, and treatment response in people with BD.

Glycogen synthase kinase 3β has been a gene of particular interest in this regard. In addition to phosphorylating multiple clock proteins and influencing circadian period length (Martinek et al., 2001), GSK3β also participates in intracellular stress signaling and neurotransmission. Elevated GSK3β expression and activity has been reported in BD patients compared to controls (Li et al., 2010). In rodent models, GSK3β inhibition produces antidepressant-like effects (Mai et al., 2002; Kaidanovich-Beilin et al., 2004), and clinically, lithium-a first-line mood stabilizer-directly inhibits GSK3β, indicating that increased GSK3β activity may drive earlier onset and mood instability in BD, and that attenuation of GSK3β signaling is one mechanism through which lithium exerts antimanic and mood-stabilizing actions.

CLOCK, one of the core transcriptional activators of the circadian machinery, is another gene of interest. The CLOCK 3111T/C single nucleotide polymorphism (SNP; rs1801260) has been associated with delayed sleep phase and higher recurrence of mood episodes in BD, with C/C genotype carriers showing a later circadian phase and increased relapses (Benedetti et al., 2003). Notably, delayed sleep phase is also observed in C-allele carriers without BD (Katzenberg et al., 1998), and meta-analyses have not found consistently supported direct association between CLOCK SNPs and BD diagnosis (Kishi et al., 2009, 2011). However, more recent work suggests that CLOCK variation can influence stress sensitivity. Gyorik et al. (2021) showed that CLOCK polymorphisms moderated depressive severity in individuals who had childhood trauma and recent psychosocial stressors, suggesting that CLOCK can influence how strongly environmental stressors desynchronize circadian timing and precipitate mood symptoms. In animal work, mice with the ClockΔ19 mutation-an altered CLOCK protein that impairs normal circadian transcription-display behavioral cycles that resemble mania, euthymia, and bipolar depression, including hyperactivity, decreased sleep need, increased rewards seeking, and subsequent depressive-like states. These oscillations are partially normalized by lithium and appear to arise from altered dopamine firing dynamics in the ventral tegmental area (Mukherjee et al., 2010; Roybal et al., 2007; Kristensen et al., 2018). These preclinical models of CLOCK-driven behavioral cycling provide mechanistic insight into the regulation of mood. CLOCK mutations produce oscillations between hyperactive and depressive-like states that parallel bipolar cycling in humans. Such models recapitulate the core clinical condition-altered sleep-wake regulation, altered rewards processing, and lithium responsiveness-making them ideal for interrogating causal pathways between circadian gene function and mood instability.

PER3 also contributes to interindividual differences in circadian timing that map onto bipolar phenotypes. PER3 polymorphisms influence chronotype and sleep timing. The PER34 allele is associated with evening preference and delayed sleep phase syndromes (Archer et al., 2003; Ebisawa et al., 2001; Nesbitt, 2018), while PER35 is associated with morningness. In bipolar cohorts, PER3 variants have been associated with illness timing and severity. Benedetti et al. (2008) showed that PER35 was associated with earlier age of onset, and earlier onset is clinically relevant because it portends a more severe and recurrent course. More recent work suggests that carriers of PER34-who tend toward eveningness and show exaggerated blue-light sensitivity-may in fact present with onset in late adolescence or early adulthood (Barlattani et al., 2024), which is consistent with epidemiologic data showing peak BD burden in the teens and twenties. Individuals with PER34 also appear unusually sensitive to blue light (Chellappa et al., 2012). That point matters because light-manipulation strategies (evening blue-light blocking, dawn or mid-day bright-light therapy) can be leveraged to stabilize sleep timing, improve insomnia, and reduce depressive symptoms in BD (Henriksen et al., 2014; Esaki et al., 2020; Ritter et al., 2021; Mead et al., 2023). PER3 genotype may therefore help explain why some bipolar patients respond so robustly to circadian/light-based interventions while others do not.

BMAL1 (ARNTL), which heterodimerizes with CLOCK to drive the transcriptional arm of the circadian loop, has also been implicated in BD. Human genetic studies have found associations between BMAL1 variation and BD, and preclinical work buttresses this finding (Mansour et al., 2005; Soria et al., 2010). BMAL1 knockout mice lose normal circadian rhythmicity in constant darkness, display fragmented and dysregulated sleep-wake cycles, accumulate slow-wave activity more rapidly (indicating high sleep pressure), and recover poorly from sleep deprivation (Laposky et al., 2005; Zielinski et al., 2017). These animals are more difficult to keep awake and show reduced baseline activity, pointing to a fundamental deficit in arousal regulation. Translationally, BD patients who are good responders to lithium show higher BMAL1 expression in neuronal tissue than non-responders (Mishra et al., 2021), and variants in ARNTL/BMAL1 have been linked to lithium’s prophylactic efficacy (Rybakowski et al., 2014). This positions BMAL1 not just as a circadian pacemaker gene but also as a potential mood stabilizer response predictor.

### Stress and trauma in HPA-axis perturbation

Psychosocial stress, and specifically trauma, is an important environmental risk factor for BD. Patients with BD have been found to experience rates of trauma significantly higher than the general population (Hernandez et al., 2013). Trauma is associated with greater symptom severity, comorbidities, and earlier onset of illness as well as more rapid cycling, frequent mood episodes, and increased risk for suicide in patients with BD (De Bellis and Zisk, 2014; Agnew-Blais and Danese, 2016). Prevalence rates for any form of trauma in patients with BD range between 50% and 80%, with up to 82% reporting cumulative stress over the course of their life (Maguire et al., 2008; Assion et al., 2009; Rowe et al., 2024). Emotional trauma-especially in childhood-is the most common subtype of trauma reported by BD patients (Dualibe and Osório, 2017). In those with early trauma, BD has been associated with earlier illness onset, rapid cycling, increased risk for psychosis, higher rates of cannabis use, and suicidality (Murphy et al., 2022). In contrast to short-term, non-traumatic stress, traumatic stress can induce long-lasting neurochemical alterations that create a new physiological set point characterized by hyperarousal and sustained activation (Weber and Reynolds, 2004). Childhood trauma has been associated with greater illness severity and severity of manic and depressive episodes in BD, comorbid PTSD and anxiety disorders, higher rates of substance use, and increased suicide attempts (Agnew-Blais and Danese, 2016; De Bellis and Zisk, 2014; Monteleone et al., 2020). In fact, as many as 55% of patients with BD may also qualify for a diagnosis of PTSD (Cerimele et al., 2017).

Genetic and epigenetic variation, which interact with trauma to influence the downstream biological response to traumatic events, is known to influence HPA-axis reactivity to stressors (Watson et al., 2007). For example, variants in FKBP5, which modulates glucocorticoid receptor sensitivity, have been associated with increased stress reactivity, suicidality, and PTSD (Brix et al., 2022; González-Castro et al., 2023). Emotional abuse and neglect have been associated with reduced DNA methylation of FKBP5 intron 7 (rs1360780) in BD (Saito et al., 2020) and in individuals with depression with a history of childhood maltreatment (Großmann et al., 2024). Although Klinger-König et al. (2019) did not observe a significant association between childhood maltreatment and FKBP5 methylation in their cohort -likely due to a limited number of individuals with documented cases of maltreatment -they did identify an overall reduction in FKBP5 methylation among those with depression. To date, few studies have examined the relationship between trauma-related FKBP5 polymorphisms or other HPA-axis gene variants in modulating BD symptom severity, cycling frequency, or treatment response.

The HPA-axis is the primary stress-response system, serving as an interface between psychological stress and endocrine and immune output. It is activated by the secretion of corticotropin releasing hormone (CRH) from the hypothalamus, which then stimulates the release of adrenocorticotropic hormone (ACTH) from the anterior pituitary gland. ACTH travels through the bloodstream to the adrenal cortex, which, in response, secretes glucocorticoids, namely cortisol (in humans). Cortisol acts through a negative feedback loop to suppress both the synthesis and secretion of CRH and ACTH from the hypothalamus and anterior pituitary, respectively. Chronic or traumatic stress can disrupt this negative feedback loop, leading to prolonged cortisol secretion and reduced feedback sensitivity, which is believed to underlie vulnerability to stress-related disorders, including BD.

Findings of HPA-axis dysregulation in BD are compelling. Multiple studies have found that patients with BD have higher hair cortisol concentration (HCC) - a measure of cumulative cortisol output over time-than healthy controls (Aas et al., 2019; Streit et al., 2016; van den Berg et al., 2020; Milo et al., 2024). In Streit et al.’s (2016) study, patients experiencing a manic episode had the highest mean HCC levels across the BD and schizophrenia cohorts, and HCC was positively correlated with YMRS scores. van den Berg et al. (2020) reported a similar finding, and a meta-analysis by Belvederi Murri et al. (2016) found that patients with BD-especially those with current mania-exhibit elevated basal cortisol and ACTH levels both in the morning and after administration of dexamethasone. CRH, on the other hand, can remain largely unaffected in BD (Girshkin et al., 2014). Furthermore, ACTH and CRH has been shown to directly increase intestinal permeability through mast-cell degranulation and the secretion of cytokines that modulate tight-junction function and gastrointestinal illnesses (Bradford et al., 2012; Ge et al., 2022; Molotla-Torres et al., 2023; Overman et al., 2012). Interestingly, exposure to adverse childhood experiences has been linked to the development of irritable bowel syndrome, as well as greater severity of gastrointestinal symptoms in affected individuals (Park et al., 2016). These findings suggest a possible mechanistic link between stress physiology and gut barrier function that may contribute to systemic inflammation, and further support the relevance of gut-brain axis studies to understanding BD pathophysiology.

Cumulative evidence also suggests that cortisol levels are elevated in BD across multiple mood states. Heightened cortisol levels have been reported in mania, depression, and mixed episodes, and other data suggest that HPA-axis dysregulation is a feature of the illness more broadly, rather than specific to a single phase of illness (Sonino et al., 1998; Herane-Vives et al., 2020; Cervantes et al., 2001; Milo et al., 2024). However, findings are not completely consistent across the literature. For example, Valiengo et al. (2012) found that plasma cortisol levels were lower in drug-naïve individuals experiencing their first manic episode, suggesting that medications may be confounding cortisol measurements in some of the other studies. Similarly, in earlier work, Joyce et al. (1987) described elevated daytime cortisol levels in depressive episodes but comparatively lower levels during mania. Conversely, patients newly diagnosed with BD - those who present with dysphoric mania -may have elevated daytime cortisol levels (Ahmed and Patil, 2024; Joyce et al., 1987). Lastly, it may also be the case that hypocortisolemia may ensue in people who’ve experienced historically elevated cortisol responses (Fries et al., 2005).

These discrepancies may reflect a dynamic and cumulative process of cortisol regulation. Measures of cumulative cortisol output, like HCC, are often found to be elevated across mood states and may represent an overall, long-term effect of HPA axis activity over time. Furthermore, stress-related subtypes of mood episodes may differentially influence cortisol levels within BD. For example, dysphoric versus euphoric mania or melancholic versus atypical depression may alter cortisol response (Valiengo et al., 2012). It is also possible that glucocorticoid administration can induce both manic and depressive symptoms, with these effects more pronounced in those with pre-existing mood vulnerability (Nasereddin et al., 2024). Similarly, in Cushing’s syndrome (CS), which is characterized by chronically high cortisol due to endocrine pathology, affective disturbances are common and most frequently present as depression but can include mania and hypomania (Sonino et al., 1998; Lin et al., 2020). This suggests that dysregulation of cortisol may be a trait-like abnormality in BD with state-dependent influences, rather than a reliable biomarker of any single mood state.

Several outstanding questions remain regarding the cortisol profile in BD. It is unclear if mania and depression are associated with distinct cortisol profiles (e.g., hyper- versus hypocortisolemia), if cortisol is elevated across mood states and has a distinct effect on specific aspects of behavior or mood depending on downstream processes (such as glucocorticoid receptor sensitivity, transport, or tissue-specific metabolism), or how cumulative cortisol levels can be high but not acutely related to symptom expression in a given mood episode. Is there some sort of buffering mechanism that delays onset or progression once a certain threshold is crossed? In other words, what distinguishes cases where higher cortisol is a state effect versus a trait effect? In a similar way, it will also be important to elucidate whether the observed differences are due to altered cortisol production, metabolism, cellular uptake, or receptor-level signaling, and whether these features differ across bipolar subtypes, illness trajectories, or trauma history. Addressing these points will be an important next step in this area. For now, it is clear that stress physiology is important, and that interventions that reduce HPA-axis activation or promote resilience may meaningfully reduce vulnerability to both manic and depressive states.

To summarize, stress and trauma have a major impact on BD by dysregulating the HPA-axis, elevating cortisol and ACTH, and reducing the body’s ability to return to baseline. Childhood trauma in particular has been associated with earlier age of onset, greater episode severity, and higher rates of suicidality (Guillen-Burgos et al., 2025). HPA hyperactivity may also promote systemic inflammation, intestinal hyperpermeability, and anhedonia, contributing to the pathological stress response (Madison and Bailey, 2024). Trauma may also interact with FKBP5 methylation status and glucocorticoid feedback sensitivity to increase vulnerability for BD in an environmentally-dependent gene × environment interaction. The HPA axis is thus an important interface between psychosocial stress, immune activation, metabolic imbalance, and mood dysregulation. Further exploration of its bidirectional crosstalk with inflammatory and gut–brain signaling may be key to the future development of interventions to increase stress resilience and mood stability in BD.

## Mitochondrial dysfunction and metabolism in bipolar disorder

Mitochondrial energy metabolism, stress responses, and brain homeostasis are fundamentally linked. Mitochondria fuel neuronal excitability and synaptic activity, synaptic plasticity, and long-term potentiation (LTP) through production of ATP, all of which enable adaptation to changing environmental demands. The metabolic hypothesis of BD argues that mitochondrial dysfunction contributes to illness development and progression through impaired oxidative phosphorylation (OXPHOS), redox and nitrosative stress response, inflammatory signaling, and stress response pathways that may also influence response to treatment and illness course (Campbell and Campbell, 2024; Khayachi et al., 2025).

Periods of high energy activation and low energy withdrawal are a hallmark of BD in clinical presentation (Campbell and Campbell, 2024). Variability in circadian timing, rest-activity cycles, and psychomotor activation, points to mismatch in metabolic supply and neuronal demand across illness episodes. Changes in energy have frequently been reported as prodromal to mood shifts: mania/hypomania is often precipitated by elevated drive and motor activation (Wright et al., 2012; Thomson et al., 2015). In bipolar depression, psychomotor slowing, fatigue, and severely reduced behavioral activation are prominent features (Malhi and Byrow, 2016). It’s proposed that these two behavioral extremes are accompanied by opposing metabolic states.

Neuroimaging and metabolomics have provided evidence of a mitochondrial inefficiency underlying this bipolar metabotype. Magnetic resonance spectroscopy (MRS), positron emission tomography (PET), and metabolomics have demonstrated patterns of impaired OXPHOS associated with increased glycolysis (Cordani et al., 2025; Lam et al., 2023; Wu C. et al., 2021; Wu Z. et al., 2021). While glycolysis can support partial compensation for mitochondrial ATP production, it is much less efficient. This phenotype has been described as a bipolar “metabotype,” a tendency to fluctuate between decreased cerebral glucose metabolism during depressive phases and increased glycolysis during manic and/or hypomanic states (Campbell and Campbell, 2024).

The alternation between these states during mania, however, may not simply reflect a switch to hyperglycolysis. Another possibility is that manic states represent a phase of increased mitochondrial metabolic demand, upregulation, and oxidative stress before cellular stress signaling is engaged. For example, work on excitotoxic neuronal injury has shown that mitochondrial hypometabolism and injury often follows an earlier phase of mitochondrial hypermetabolism, a period in which energetic demand and oxidative stress accrues (Naia et al., 2023; Sercel et al., 2024). This model is consistent with FDG-PET studies that have shown reduced cerebral glucose uptake during depression and elevated uptake during mania (Baxter et al., 1985). Additional evidence for altered bioenergetics comes from reports of elevated lactate (a product of glycolysis) in the ACC, caudate, and CSF of BD patients (Dager et al., 2004; Kim D. J. et al., 2007; Regenold et al., 2009; Chu et al., 2013; Weber et al., 2013; Scaini et al., 2020). Accumulation of lactate with concomitant acidosis indicates impaired oxidative metabolism.

Metabolic inefficiency also has downstream consequences on electrophysiological function. Glycolysis alone is not sufficient to support the ATP demands of the Na^+^/K^+^-ATPase that maintains resting membrane potential. As ATP becomes depleted, intracellular Na^+^ accumulates, intracellular K^+^ is lost, and neurons enter a state of chronic depolarization (Shrivastava et al., 2020). Chronic depolarization leads to opening of voltage-gated Ca^2+^ channels, causing influx of Ca^2+^ and excitotoxic signaling cascades that can result in cellular injury and apoptosis. This provides one mechanism by which mitochondrial dysfunction could contribute to neuroprogression in BD (Lam et al., 2023).

Glutamate metabolism is another rapid metabolic resource that can be employed during cognitively and emotionally demanding conditions (Kinoshita et al., 2016; Wiehler et al., 2022). This appears to be especially true in the PFC and ACC (Chen C. H. et al., 2010; Chen G. et al., 2010), structures that are central to executive function, salience processing, and emotion regulation. In BD, it is possible that this glutamate-dependent energy system becomes dysregulated. Consistent with hypermetabolism, manic and mixed states have been associated with hyperglycolysis with increased glutaminolysis (breakdown of glutamine for energy production), whereas bipolar depression is characterized by hypometabolism with reduced glycolysis and lactate production (Campbell and Campbell, 2024). Interestingly, increased brain lactate and low intracellular pH have been observed across the depression, mania, hypomania, and even euthymic states (Brady et al., 2012; Machado-Vieira et al., 2017), suggesting that mitochondrial dysfunction may be a trait-like feature of BD, rather than a state-dependent abnormality.

Magnetic resonance spectroscopy has been widely used to probe Glu and Gln *in vivo*. At lower magnetic field strengths, these metabolites are often difficult to separate in spectra and are commonly reported as a single Glx variable. Glu and Gln cycle through tightly coupled neuronal-astrocytic metabolic pathways, in which synaptically released glutamate is taken up by astrocytes and converted to glutamine, which is recycled back to neurons. Thus, disruption of this cycle serves as an indirect measure of disturbed neuron-glia metabolic coupling. Findings across BD studies have been mixed, however, and depend on mood state, brain region, medication status, and imaging field strength.

Among cortical areas, the ACC has been a region of particular interest in BD (Strakowski et al., 2012; Campbell and Campbell, 2024). Functionally, the ACC integrates information about affective salience, cognitive control, decision-making, and impulse regulation. Resting ACC hyperactivity during mania has been shown to correlate with symptom severity and is related to increased emotional reactivity, intrusive cognition, and compulsive or perseverative behaviors (Drevets et al., 1997; Rubinsztein et al., 2001; Ghaznavi and Deckersbach, 2012). During bipolar depression, ACC activity is often lower. Notably, during tasks involving recognition of emotional information, manic patients may show blunted ACC activation, potentially underlying lack of awareness of social distress signals (Lennox et al., 2004).

The possibility has been raised that increased glutaminolysis in the ACC represents a putative “metabolic signature” of BD (Needham et al., 2023; Campbell and Campbell, 2024). In the setting of inefficient mitochondrial OXPHOS, neural metabolism can shift toward glycolysis and glutamine utilization to produce ATP (Shulman et al., 2001). In TBI, mitochondrial Ca^2+^ overload causes OXPHOS inefficiency and an acute energy crisis, triggering compensatory hyperglycolysis and glutamate release to fuel Na^+^/K^+^ pump activity (Bergsneider et al., 2001; Gruenbaum et al., 2022; Mira et al., 2023; Vespa et al., 1998). It has been proposed that BD may represent a lower intensity, chronic version of a similar metabolic shift between hypometabolism and compensatory hyperactivation.

If increased glutaminolysis is a primary driver of mania, one would expect to see increases in glutamate consistently across manic states in mood-relevant brain regions. The data do not yet support this pattern. In a meta-analysis of 40 studies (1,135 BD patients; 964 controls), Ino et al. (2023) found that ACC Glx levels were elevated in BD overall, but that this effect was driven almost entirely by bipolar depression, rather than mania. Studies that were acquired at field strengths > 1.5 Tesla also report an increase in Gln relative to Glu, suggesting increased astrocytic conversion of glutamate to glutamine during depression (Öngür et al., 2008; Yüksel and Öngür, 2010). Glutamine elevation would therefore be more consistent with bipolar depression, whereas glutamate increases during mania are less consistently observed. This complicates models in which hyperglutaminolysis is a unifying mechanism for manic pathophysiology.

Medication exposure is a particularly significant confound in this literature. The majority of spectroscopy studies include medicated patients, and very few include unmedicated manic patients, making it difficult to interpret group differences. The limited data from small studies of unmedicated patients suggest that unmedicated depressed patients have lower CSF glutamate relative to controls (Frye et al., 2007a,b) and that unmedicated bipolar depressive patients show increased CSF glutamine (Levine et al., 2000). A meta-analysis of only non-medicated adults (five studies) found increased Glx and/or glutamate in multiple regions across different mood states - including ACC in depression, whole brain in depressed or mixed states, dlPFC in multiple mood states, occipital cortex in euthymia, and dlPFC in mania (Gigante et al., 2012). In largely unmedicated manic patients scanned at 1.5 Tesla, elevated Glx (reported as glutamate) was observed in the dlPFC (Michael et al., 2003; Gigante et al., 2012). However, it is not clear from these studies whether the Glx signal is dominated by Glu or Gln at this field strength (Auer et al., 2000; Michael et al., 2003; Snyder and Wilman, 2010; Ino et al., 2023). Disentangling these uncertainties, especially in unmedicated and state-stratified cohorts, will be an important goal for future studies.

### Mitochondrial defects as a predisposition toward impaired metabolism

In addition to shifts in fuel use (glycolysis vs. oxidative phosphorylation), BD has been linked to a more direct inhibition of the mitochondrial machinery. In line with the aforementioned changes in fuel metabolism, several studies have implicated a downregulation of mitochondrial enzymes of the Krebs (citric acid) cycle and the ETC in BD, in both human and manic-mimetic animal models.

Manic-like states in rodents have been induced using a variety of psychostimulants, most commonly d-amphetamine, methamphetamine or fenproporex. As described in the previous section, these models typically result in a constellation of behaviors thought to be analogous to mania (hyperactivity, hypermotility, impulsivity, aggression, etc.). The induction of these behaviors is coupled with a global suppression of mitochondrial function. A consistent finding across these studies is a decrease in the activity of several Krebs cycle enzymes (citrate synthase, succinate dehydrogenase, malate dehydrogenase) as well as inhibition of ETC complexes I–IV and of creatine kinase in the prefrontal cortex, hippocampus, amygdala and striatum (Corrêa et al., 2007; Streck et al., 2008; Valvassori et al., 2010; Feier et al., 2013; Rezin et al., 2014). To take one example, a low dose of methamphetamine (0.25 mg/kg) was found to decrease the activity of citrate synthase in the prefrontal cortex, amygdala, hippocampus and striatum while also inhibiting succinate dehydrogenase, malate dehydrogenase, and ETC complexes I, II, II–III, and IV in these same regions (Feier et al., 2013). Fenproporex administration results in a similar suppression of mitochondrial activity, decreasing the activity of citrate synthase, malate dehydrogenase, and succinate dehydrogenase (Rezin et al., 2014). These findings indicate an overall limitation of oxidative ATP production from multiple points in the mitochondrial cascade.

There is also some evidence that energy buffering capacity is similarly affected. Creatine kinase is a key enzyme in the buffering of ATP in tissues that require a high energy turnover, as it allows for the rapid conversion of phosphocreatine to ATP (virtually instantaneously, with a rate that is faster than diffusion). In manic-like animal models of BD, ouabain-induced hyperactivity has been shown to induce a long-term decrease in creatine kinase activity in the hippocampus and other brain regions (Streck et al., 2008; Freitas et al., 2010). As such, the reduction of creatine kinase may also play a role in restricting the brain’s capacity to buffer its energetic state and thus hastening the energetic decline that typically follows periods of extended hyperactivity.

Molecular analyses of human tissue converge on a similar energetic state. Analysis of transcriptomic datasets has revealed a downregulation of creatine kinase and a general trend of downregulated nuclear-encoded mitochondrial genes, including several Krebs cycle enzymes as well as ETC complex subunits (MacDonald et al., 2006). Clinical studies have replicated these findings using both microarray and qPCR approaches to demonstrate a decrease in mRNA and protein levels for several mitochondrial respiratory chain components, lower activity of the ETC complexes, and decreased activity of several Krebs cycle enzymes in BD brain tissue (MacDonald et al., 2006). Andreazza et al. (2010) found that complex I activity was significantly lower in prefrontal cortex tissue of depressed BD patients, and also found decreased expression of the complex I subunit gene NDUFS7. However, the activity of complex I was only mildly decreased in peripheral blood mononuclear cells of euthymic patients, which may indicate some degree of mood-state specificity (Andreazza et al., 2010; Gubert et al., 2013). Of course, medication use may confound these measurements to a certain extent.

## Immune system dysfunction and inflammation

Inflammatory abnormalities have been consistently reported in BD and evidence has accumulated implicating immune dysregulation in its pathophysiology (Futtrup et al., 2020; Jones et al., 2021; Sayana et al., 2017). As noted above, many studies document elevated circulating cytokines in BD patients relative to healthy controls, indicative of increased inflammatory signaling activation.

One of the most widely studied of these markers is interleukin-1β (IL-1β), which is produced primarily by activated macrophages and microglia and is an upstream mediator of inflammatory signaling cascades. IL-1β has been observed at elevated concentrations during mania relative to depression (Ortiz-Domínguez et al., 2007), and higher serum IL-1β levels are associated with increased suicide risk in BD (Monfrim et al., 2014; Dickerson et al., 2017). Supporting the notion that there is some degree of regulatory compensation, the interleukin-1 receptor antagonist (IL-1Ra), an endogenous factor that blocks IL-1β signaling, is also elevated during mania and partial remission but normalizes after full remission (Modabbernia et al., 2013; Goldsmith et al., 2016). This pattern suggests that inflammatory activation may track with mood state and partially normalize during recovery.

Similar patterns have been reported for interleukin-6 (IL-6), which is another cytokine that is frequently elevated in BD (Goldsmith et al., 2016; Kauer-Sant’Anna et al., 2009; Kim Y. K. et al., 2007; Pantović-Stefanović et al., 2016). Experimental studies have provided some mechanistic context for this finding: IL-6 elevation renders individuals more vulnerable to the effects of stress and increases depression-like behaviors in animal models of depression, while IL-6 knockout models are stress-resistant (Hodes et al., 2016). In clinical populations, IL-6 concentrations often normalize with treatment, which is consistent with the possibility that inflammatory activation tracks with illness activity and therapeutic response (Kim Y. K. et al., 2007; Pantović-Stefanović et al., 2016).

Tumor necrosis factor-α (TNF-α) represents another inflammatory signal elevated in BD (Kauer-Sant’Anna et al., 2009). TNF-α elevations are frequently reported in BD and can contribute to a state of sustained inflammatory signaling by inducing additional cytokines and reactive oxygen species (Jones et al., 2021). Elevated TNF-α has been associated with poor response to lithium treatment (Guloksuz et al., 2012) and has also been linked to thyroid abnormalities that are commonly observed in lithium-treated patients (Barbesino, 2010; Lee et al., 1992; Shine et al., 2015; Joffe, 2002). Notably, not all studies have reported differences between BD patients and healthy controls (Hung et al., 2007), a finding that could be explained by the anti-inflammatory effects of some antidepressant medications (Brustolim et al., 2006; Brymer et al., 2019; Patel et al., 2023). There is also evidence that the inflammatory profile of BD may vary across stages of the illness. For example, some studies suggest that TNF-α elevations become more pronounced in later stages of BD and are associated with impairments in inhibitory control, aggression, and altered ACC activity (Barbosa et al., 2012; Barzman et al., 2014). Animal work has provided additional support for a role for TNF signaling in BD behavior: TNF receptor knockout models are protected from stress-induced aggression and agitation (Patel et al., 2010).

Systemic inflammatory activity is also indexed by the elevation of C-reactive protein (CRP), an acute-phase protein that is induced by IL-1 and IL-6. Medication-free BD patients have markedly higher levels of CRP during manic episodes relative to healthy controls, and CRP levels are reduced following treatment (Uyanik et al., 2015). Although CRP levels are elevated during all mood states (Cunha et al., 2008), they appear to be most robust during mania (Tsai et al., 2014; Fernandes et al., 2016; Poletti et al., 2024). Furthermore, increased CRP has been linked to altered functional connectivity in rewards circuitry, as well as to trauma exposure, a known environmental risk factor for BD (Fagundes et al., 2013; Mehta et al., 2020). For these reasons, high-sensitivity CRP (hs-CRP) has emerged as a useful biomarker of treatment response in unmedicated BD patients.

The inflammatory profile observed in BD may help to explain the disorder’s extensive medical comorbidity. In addition to frequently co-occurring with other psychiatric disorders (Yapici Eser et al., 2018; Friìas et al., 2016), BD is also associated with an elevated risk for cardiovascular, cerebrovascular, and metabolic diseases (Correll et al., 2017; Dalkner et al., 2021; Jones et al., 2021). These overlaps are likely not coincidental but rather reflect shared inflammatory and metabolic vulnerabilities. Several lifestyle factors that are commonly associated with BD, including smoking, sleep disruption, and substance use, may further amplify inflammatory signaling (Salloum and Brown, 2017; Grunze et al., 2021; Dalkner et al., 2021). Indeed, more than 90% of individuals with BD experience at least one lifetime comorbidity, and over 70% of individuals with BD have three or more comorbidities (Merikangas et al., 2007).

Chronic immune activation may contribute to neural injury by promoting a state of sustained cytokine signaling, oxidative stress, and glial reactivity. Notably, inflammatory bias within the brain can also have broad metabolic consequences by reshaping cellular energy metabolism. Pro-inflammatory signaling can drive excessive mitochondrial activity and oxidative stress, which may ultimately compromise mitochondrial efficiency and result in a compensatory shift toward hyperglycolytic metabolism at the expense of oxidative phosphorylation (Esteves et al., 2023). It is important to note that this relationship is bidirectional: mitochondrial dysfunction can itself conditionally amplify pro-inflammatory signaling, which establishes a reinforcing feedback loop between metabolic stress and immune activation (Ball et al., 2025; Gazestani et al., 2023; Naia et al., 2023). This immune-driven metabolic reprogramming can have profound effects on neuronal energetics and synaptic stability, a pattern also observed in several neurodegenerative conditions and one that may help to explain the elevated risk for later-life neurodegenerative disease among individuals with BD (Esteves et al., 2023; Gazestani et al., 2023; Naia et al., 2023). This process has been implicated in gray matter loss, white matter microstructural abnormalities, and cognitive decline in BD (Goldsmith et al., 2016; Wakonigg Alonso et al., 2024). Persistent low-grade inflammation therefore appears to function not only as a biomarker of disease activity but also as a potential driver of illness progression and the high burden of systemic comorbidity that is observed in BD.

## Gut-brain axis and microbiome interactions in bipolar disorder

The gut microbiota, the collective term for the trillions of microorganisms residing in the gut, is increasingly thought to play a role in the neuroimmune and metabolic dysregulation that characterizes BD (Donaldson et al., 2016; Nikolova et al., 2021; Jones et al., 2021). Changes in the composition and function of the gut microbiota can affect a variety of physiological processes, including immune cell signaling, metabolic homeostasis, and intestinal epithelial barrier function (Weiss and Hennet, 2017; Spadoni et al., 2015). These interactions between the gut and the rest of the body can lead to inflammation and changes in neuroendocrine function.

BD has been associated with altered gut microbial communities in several studies. Reduced microbial richness and diversity, including the depletion of Firmicutes and Actinobacteria phyla as well as a reduction in Faecalibacterium, Ruminococcaceae family, and other short-chain fatty acids (SCFAs) producing bacteria have been reported in BD patients (Painold et al., 2019). Loss of SCFA-producing bacteria has been associated with an altered intestinal barrier (Pérez-Reytor et al., 2021), which can lead to increased translocation of microbial products across the intestinal membrane and systemic circulation of inflammatory cytokines (Ghosh et al., 2020) - both have been reported in BD (Couce et al., 2023; Nguyen et al., 2018; Pinzi et al., 2025). BD patients have also been reported to have increased Flavonifractor and Enterobacteriaceae family, both of which are Gram-negative bacteria known to produce lipopolysaccharide (LPS) (Coello et al., 2019; Lu et al., 2019). The former also correlated with increased oxidative stress and body mass index (BMI). Both LPS-producing bacteria expansions are associated with a decreased Bifidobacteria to Enterobacteriaceae (B/E) ratio, a proposed biomarker of gut dysbiosis (Lai et al., 2025). This is similar to the inflammatory microbiome alterations observed in metabolic syndrome, a well-known comorbidity of BD (Ramírez-Ortiz and Girón, 2023; Yamagata et al., 2017).

**FIGURE 1:**
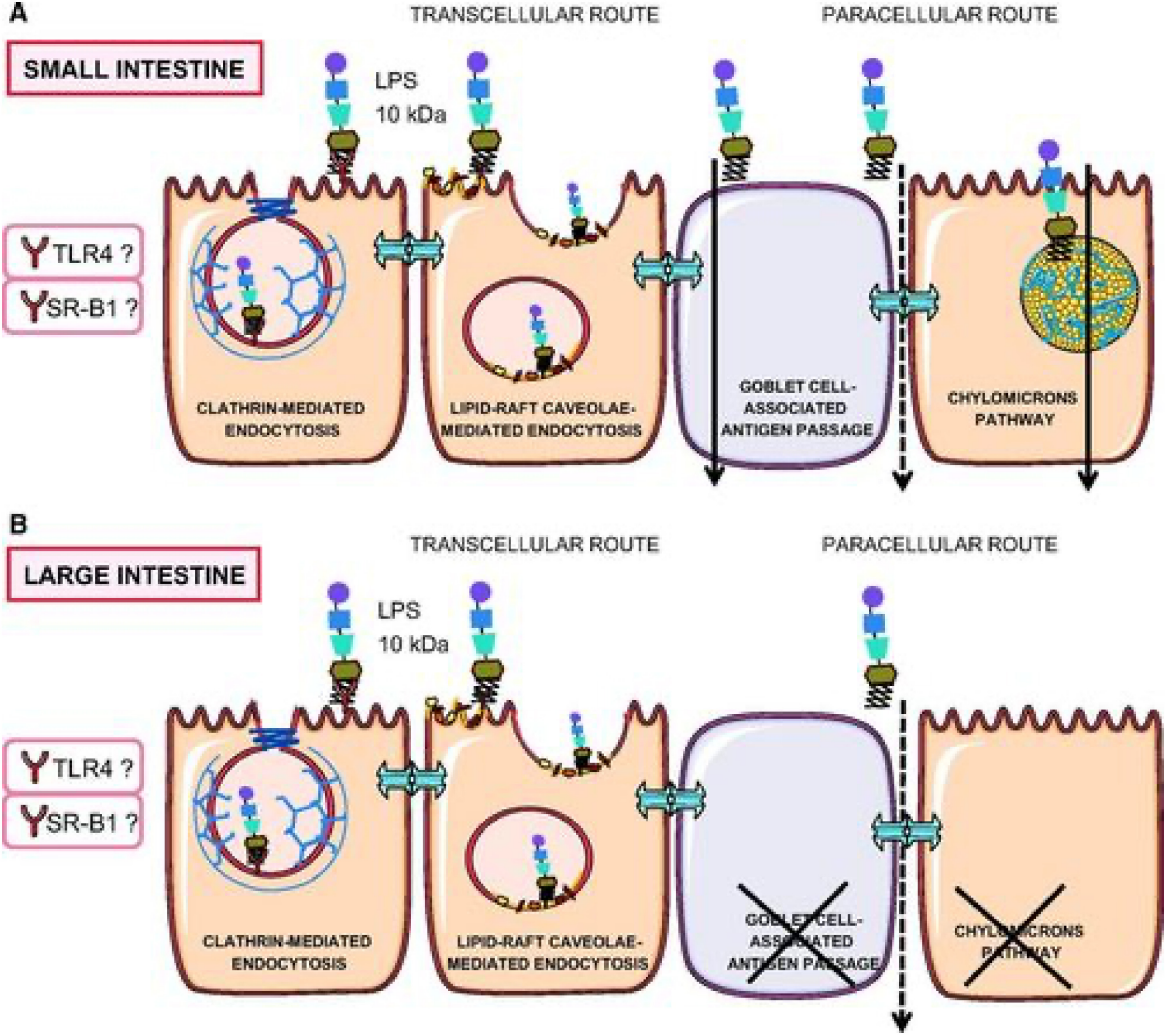
Intestinaland colonic endotoxin absorption. Endotoxin absorption is generally negligible in healthy individuals and occurs primarily in those with conditions that increase intestinal permeability (e.g., trauma, pancreatitis). Shown here are the principal pathways of LPS uptake across the small intestine **(A)** and large intestine **(B)**. The figure was made by - and reproduced from - Guerville and Boudry (2016).

These gut microbial changes are thought to disrupt the microbiota-gut-brain (MGB) axis, a bidirectional communication network connecting the gut microbiota to the central nervous system (CNS) (Chakrabarti et al., 2022). This axis includes neural, endocrine, and immune pathways through which the intestinal microbial can impact CNS (Nikolova et al., 2021). The MGB axis is frequently dysregulated in BD. For example, higher corticosterone levels and disrupted diurnal cortisol rhythm, two alterations frequently reported in BD, can themselves induce changes in gut microbial composition and intestinal permeability (Figure 1; Mukherji et al., 2015; Vegiopoulos and Herzig, 2007). Stress-induced HPA-axis activation also leads to increased intestinal permeability through mast-cell degranulation and cytokine release (Molotla-Torres et al., 2023). This creates a vicious cycle in which psychological stress can alter the gut microbial community and vice versa.

Patients with microbiome abnormalities also show biochemical signs of intestinal barrier dysfunction (Schreiber et al., 2024). Circulating levels of serum immunoglobulins to commensal bacteria, LBP/LPS and zonulin, indicators of microbial translocation and impaired epithelial barrier function, have been found to be higher in BD patients (Kılıç et al., 2020; Simeonova et al., 2020), as well as anxiety and depression (common features of BD) (Stevens et al., 2018). IL-6, TNF-α, and CRP were also found to be increased, further supporting the idea that microbial translocation can contribute to the low-grade systemic inflammation present in BD (Caradonna et al., 2000; Papa et al., 2023). This could also, at least partially, explain the strong comorbidity of BD with metabolic and cardiovascular conditions (Dalkner et al., 2021).

BD is often associated with environmental and lifestyle factors that can also adversely affect gut microbial communities (Evans et al., 2017). Stress, sleep disruption, poor diet, and long-term exposure to psychotropic medications are all known to influence gut microbial composition in BD (Durgan et al., 2019; Nikolova et al., 2021). Many of these factors can result in overgrowth of LPS-producing Gram-negative Proteobacteria (Weiss and Hennet, 2017). In turn, chronic microbial exposure to LPS can lead to persistent TLR4 signaling in epithelial and immune cells (Zhan et al., 2022). This, in turn, leads to prolonged secretion of inflammatory cytokines such as TNF-α, IL-6, and IL-1β (Caradonna et al., 2000), which can impair the veracity of the intestinal lining (Al-Sadi et al., 2016; Andrews et al., 2018). The aforementioned pro-inflammatory cytokines are also commonly increased in BD and contribute to the disorder’s pathophysiology and neuroprogression (Jones et al., 2021).

Alterations in intestinal barrier function may represent a critical mechanism through which gut microbiota dysregulation promotes systemic inflammation in BD. Dysbiosis is linked to decreased expression of tight-junction proteins including occludin (OCLN), zonula occludens-1 (ZO-1), and claudin-3, thereby compromising mucosal barrier integrity (Papa et al., 2023; Singh et al., 2019). Heightened permeability enables microbial metabolites and endotoxins to enter systemic circulation, driving oxidative stress, endothelial dysfunction, and neuroinflammatory signaling. Consistent with this hypothesis, BD patients show elevated levels of zonulin and LBP/LPS in circulation, which are markers of intestinal barrier dysfunction and immune activation (Khusainova et al., 2024).

Experimental and clinical evidence also implicates restoration of microbial homeostasis in partially mitigating these inflammatory pathways. Supplementation with *Akkermansia muciniphila*, a mucin-degrading bacterium known to support epithelial barrier function, promotes tight-junction expression and attenuates inflammatory cytokine production in preclinical models (Bian et al., 2019; Zheng et al., 2023). Similarly, probiotic administration of *Bifidobacterium* and *Lactobacillus* strains have been shown to reduce intestinal permeability and systemic inflammation by enhancing production of anti-inflammatory mediators such as interleukin-10 (IL-10) and Peroxisome proliferator-activated receptor gamma (PPAR-γ), and by suppressing pro-inflammatory signaling molecules and pathways such as TNF-α and IL-1β (Chorawala et al., 2021; di Vito et al., 2022; Haque et al., 2024; Ruiz et al., 2017). In early clinical trials using probiotic formulations containing these taxa, improvements in mood symptoms, cognitive performance, and attentional function have been reported in individuals with BD, although larger controlled trials are needed to confirm these preliminary findings (Dalkner, 2018; Dickerson et al., 2018; Eslami Shahrbabaki et al., 2020).

In addition to its direct gastrointestinal effects, increased intestinal permeability may also promote systemic immune activation and metabolic dysfunction in BD. Heightened levels of circulating LPS and pro-inflammatory cytokines may contribute to mitochondrial dysfunction, synaptic plasticity deficits, and white-matter abnormalities, thereby linking gut-derived inflammation to neuroprogressive processes thought to underlie disease (Goldsmith et al., 2016). Similar permeability-associated inflammatory processes have been implicated in the pathophysiology of many comorbidities common to BD, including cardiovascular disease, insulin resistance, and obesity (Dalkner et al., 2021; Lewis and Taylor, 2020). Increased levels of anti-Saccharomyces cerevisiae antibodies (ASCA) and soluble CD14 (sCD14) are observed in BD patients and are indicative of immune activation in response to bacterial and fungal products (Severance et al., 2013, 2014). Additional autoimmune antibodies have been reported in BD including anti-tissue transglutaminase (anti-tTG) and anti-gliadin IgG. Anti-tTG levels are increased in manic states and decrease in treated euthymic patients while anti-gliadin IgG is increased in manic patients relative to controls (Dickerson et al., 2012; Rudzki and Szulc, 2018). The observation that these antibodies normalize with treatment suggests that intestinal immune activation may ebb and flow with illness. Inflammatory signaling pathways may further potentiate the effects of dysbiosis and increased intestinal permeability. Cytokines such as TNF-α and IL-1β have been shown to directly disrupt epithelial cell junctions, thus amplifying permeability and perpetuating the cycle of dysbiosis, inflammation, and immune activation (Al-Sadi and Ma, 2007; Jin and Blikslager, 2020). Such feedback loops may contribute to increased rates of metabolic, cardiovascular, and autoimmune comorbidities in BD (Sylvia et al., 2015; Dalkner et al., 2021).

Overall, available evidence supports a model whereby gut dysbiosis and increased intestinal permeability contribute to BD pathophysiology by inducing a state of chronic immune activation, oxidative stress, and neuroinflammatory signaling. Markers of intestinal permeability - such as LPS, zonulin, and soluble CD14 - as well as immune-reactive antibodies such as ASCA and anti-gliadin are elevated in BD and further support the relationship between intestinal barrier dysfunction and mood instability. Through its effects on HPA-axis regulation, neurotransmitter systems, and metabolic homeostasis, the gut–brain axis therefore emerges as a key pathway through which environmental exposures and genetic predispositions may intersect in the pathogenesis of BD. As such, therapeutic strategies aimed at restoring microbial homeostasis - including probiotics, prebiotics, dietary modifications, and fecal microbiota transplantation (FMT) - hold promise for adjunctive treatment and for mitigating the systemic disease burden associated with BD.

## State- and trait-specific neural circuit alterations in bipolar disorder

### An overview of neuroinflammation and barrier dysfunction in BD

BD is beginning to be conceptualized as a neuroinflammatory disorder marked by immune activation and alterations in barrier function (such as BBB breakdown) (Wakonigg Alonso et al., 2024). Post-mortem analysis reveals an upregulation of pro-inflammatory cytokines and junctional proteins in BD (Wakonigg Alonso et al., 2024).

Changes in the cerebrospinal fluid albumin quotient (QAlb)–a ratio of albumin in CSF to serum - is a commonly used metric for assessing barrier permeability. QAlb has been shown to be elevated in BD compared with healthy controls with a greater increase seen in BD I compared with BD II and correlations with number of lifetime psychotic episodes (Wakonigg Alonso et al., 2024; Zetterberg et al., 2014). However, these increases were more marked among individuals receiving antipsychotic medications, suggesting pharmacological effects on endothelial or glial barrier regulation may partially underlie these differences (Elmorsy et al., 2014).

Inflammatory signaling can also lead to changes in endothelial adhesion. Cell adhesion molecules, such as intercellular adhesion molecule-1 (ICAM-1) and vascular cell adhesion molecule-1 (VCAM-1), which are known to regulate leukocyte trafficking across vascular endothelium, are also expressed by neurons, astrocytes, and microglia. Inflammatory cytokines upregulate ICAM and VCAM to facilitate immune cell migration into neural tissue. Increased ICAM levels have been found in BD across mood states and illness subtypes with higher levels observed in those with longer illness duration (Müller, 2019; Schaefer et al., 2016). ICAM and VCAM have also been shown to increase during manic episodes compared to remission or control conditions (Turan et al., 2014). However, other studies have shown higher ICAM and lower soluble VCAM-1 during bipolar depression and MDD (Pantovic-Stefanovic et al., 2024). These results are in line with experimental findings showing that cytokines such as IL-1 and TNF-α upregulate adhesion molecule expression, leading to leukocyte infiltration and neuroinflammatory signaling (Angrand et al., 2021).

Although much of this evidence for neuroinflammation in BD is based on peripheral biomarkers or post-mortem data, *in vivo* imaging and neuropathological data are beginning to fit this same framework. A limitation of the existing literature is that very few studies assess multiple dimensions of inflammation - circulating cytokines, glial activation, peripheral immune cell infiltration, and others - in the same cohort, making it difficult to understand the causal pathways involved (Giridharan et al., 2020). However, the available data point toward a model in which chronic inflammatory signaling contributes to neuronal damage, synaptic loss, and cognitive impairment in BD (Ogłodek et al., 2026).

Microglial activation is a key aspect of this process. Chemokines such as monocyte chemotactic protein-1 (MCP-1) can promote microglial proliferation and peripheral monocyte infiltration into neural tissue. MCP-1 has been found to be elevated in the CSF of BD patients and has been associated with synaptic dysfunction and neurodegenerative processes (Zetterberg et al., 2014). A systematic review of 13 studies found consistent elevation of MCP-1 in BD, with a particularly robust effect during depressive episodes, pointing to a possible state-dependent fluctuation in microglial activation (Misiak et al., 2020; Isgren et al., 2022). Microglial priming has also been demonstrated in depressed suicide victims, and was associated with an increase in perivascular macrophage density in white matter, suggestive of persistent immune activation (Torres-Platas et al., 2014). Evidence of glial involvement in BD is further supported by elevated serum S100B levels - a marker of astrocyte activity - observed during both manic and depressive episodes but not in euthymic patients, alongside increased superoxide dismutase activity and elevated markers of lipid peroxidation regardless of mood state, suggesting a pattern of oxidative stress and astrocyte dysfunction that tracks with active phases of illness.

Neuroimaging studies further support glial activation in BD. The mitochondrial translocator protein (TSPO), which is upregulated in activated microglia and astrocytes, can be imaged using positron emission tomography (PET). Studies using [^11C]-(R)-PK11195 have reported increased TSPO binding in the hippocampus of BD patients (Haarman et al., 2016). Given the hippocampus’s central role in stress regulation and memory function, inflammatory activity in this region may further contribute to HPA-axis dysregulation, a recurrent feature of BD (Cole et al., 2022; Frodl and O’Keane, 2013).

### Structural imaging

BD has been associated with changes in brain morphology and function, although the findings are mixed across studies and methodologies. The most consistently replicated morphometric changes to date are increases in lateral ventricular volume. Abé et al. (2022) conducted a meta-analysis across 1,232 participants, and found that BD patients had larger ventricular volumes than healthy controls. The authors also found that the number of lifetime manic episodes was associated with progressive cortical thinning of the prefrontal cortex, providing evidence that manic episodes may lead to cumulative structural remodeling of frontal networks over time. However, it is difficult to determine from this work whether these changes are the result of repeated mood episodes, or if these abnormalities are preexisting neurobiological vulnerabilities that increase risk for manic recurrence and cortical atrophy.

In any case, additional convergent structural abnormalities have been described in other work. Okada et al. (2023) found larger lateral ventricular volumes and smaller hippocampi in a sample of 235 BD patients, with larger effect sizes in BD I compared to BD II. Manelis et al. (2024) also reported ventricular enlargement in a large and predominantly female sample, with the largest changes in patients with longer illness duration and more depressive symptoms. In this work, ventricular expansion was associated with lower myelination in regions close to the lateral ventricles, including proximal cingulate, frontal, sensorimotor, and right insular cortices - regions that have been associated with emotional and cognitive control in previous work. Myelination was found to be increased in more distal regions of the brain, and the authors proposed that this may represent a compensatory adaptation to localized demyelination in regions near the ventricles. Notably, loss of hippocampal volume and more global gray matter atrophy have also been observed in lithium-naïve and unmedicated individuals (Drevets et al., 1998; Hajek et al., 2012; Bearden et al., 2008; Roda et al., 2015; Okada et al., 2023), suggesting that these structural abnormalities are intrinsic to the disorder rather than a result of medication exposure. These morphological findings are consistent with a broader pattern of inflammatory vulnerability: BD patients have also been found to have a higher prevalence of white matter hyperintensities (WMH) compared to healthy controls, implicating inflammatory signaling in structural brain changes (Silva et al., 2024). Together, these structural changes likely reflect the cumulative effects of chronic inflammation, oxidative stress, and impaired neuroplasticity that accompany recurrent mood episodes.

### Functional imaging

**FIGURE 2:**
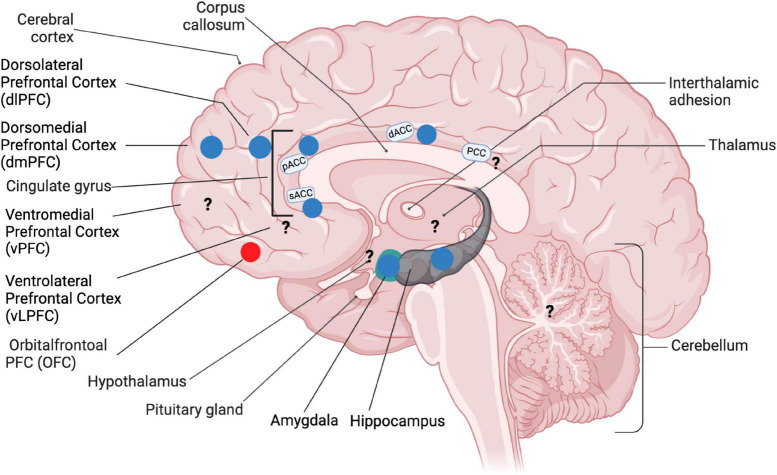
Bipolar mania and affective labeling. During affective labeling tasks, the dorsolateral prefrontal cortex (dlPFC) frequently exhibits increased activity. Similar patterns have been reported in the ventromedial prefrontal cortex (vmPFC), although findings are heterogeneous across studies, with some reports indicating reduced activation. In contrast, the ventrolateral prefrontal cortex (vlPFC) more consistently demonstrates reduced activity, while limbic regions generally show increased activation. Red markers denote regions of relatively increased activity, blue markers indicate reduced activity, and question marks highlight areas where findings remain inconsistent or insufficiently characterized with respect to mood state or task conditions.

Functional neuroimaging findings also support aberrant limbic regulation as a mechanism in emotional dysregulation in BD. Commonly, these studies report aberrant connectivity between prefrontal regulation regions and limbic emotional salience regions. Manic participants display reduced functional connectivity between the ventrolateral PFC (vlPFC) and the amygdala, thought to reflect decreased top-down regulation of emotional reactivity (Foland et al., 2008). These changes are also associated with amygdala hyperactivation and under-recruitment of the vlPFC during emotional labeling (Figure 2; Foland et al., 2008). This pattern of heightened amygdala reactivity with decreased cortical recruitment is thought to indicate a failure of cortical regulation regions to properly modulate limbic reactivity. Additionally, while reactivity to emotional stimuli is heightened in mania, patients often exhibit dampened responses to the valence/intensity of these emotional stimuli (Foland et al., 2008). Such findings may indicate an alteration in regional perfusion or inefficient processing of emotional salience (Foland et al., 2008). These findings in fronto-limbic regulation are consistent with the increased reactivity, impulsivity, and reduced empathic abilities observed during mania (Chen et al., 2006).

In this regard, these limbic regulatory abnormalities appear to be not only state-dependent but also persistent during euthymia (Ahmed et al., 2023). This may point to altered emotion-regulation circuitry as a trait-level vulnerability in BD. Reduced functional connectivity between the amygdala and ACC has also been found (Brady et al., 2017). There is also evidence for increased activation of the cingulate gyrus and SFG/dlPFC during emotional processing in (hypo)manic individuals while no differential activation in the same regions is observed in bipolar depression (Chen et al., 2006).

**FIGURE 3:**
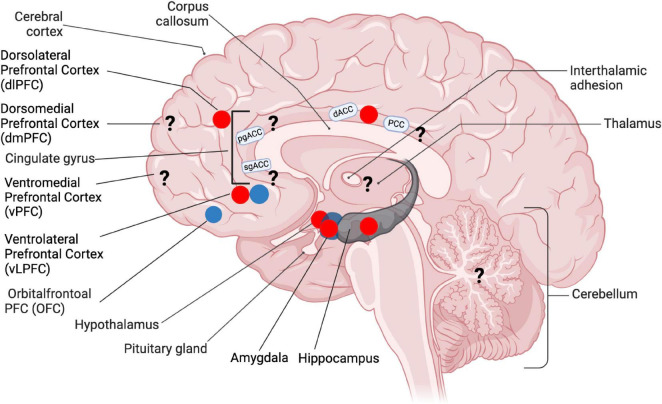
Bipolar depression and affective labeling. During affective labeling tasks, the dorsolateral prefrontal cortex (dlPFC), dorsomedial prefrontal cortex (dmPFC), cingulate gyrus, amygdala, pituitary, and hippocampus generally exhibit reduced activity. In contrast, the orbitofrontal cortex (OFC) demonstrates relatively increased activity during these tasks. Red markers denote regions of increased activity, blue markers indicate reduced activity, and question marks highlight areas where findings remain limited, inconsistent, or insufficiently characterized with respect to mood state or task conditions.

Opposite to this hyper-reactive limbic circuitry seen in mania, bipolar depression appears to involve a hypoactive regulatory cingulate network (Figure 3). Reductions in dorsal ACC perfusion with relative hyperactivity of ventral-rostral regions has been found (Pizzagalli et al., 2001). This region is thought to be related to cognitive/emotional integration and is associated with major depression responsive to treatment. A recent meta-analysis supports this finding by reporting decreased ACC perfusion in bipolar depression during affective labeling (Ahmed et al., 2023). In keeping with these findings, there is also evidence for decreased amygdala activation during emotion labeling in bipolar depression (Torre and Lieberman, 2018) and treatment-resistant depression (Ferri et al., 2017).

**FIGURE 4:**
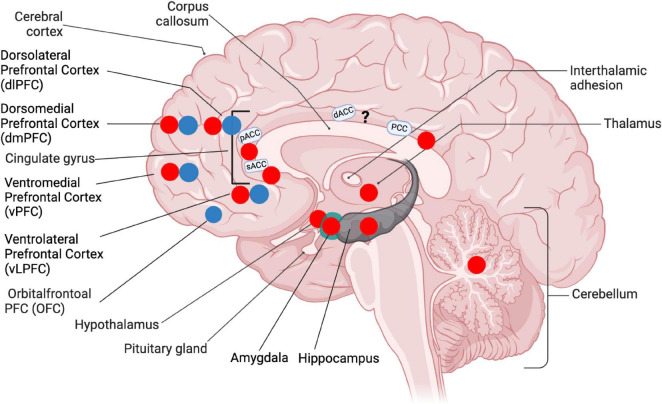
Bipolar mania at rest. This schematic illustrates regional patterns of brain activity associated with bipolar mania under resting-state conditions. Activity within prefrontal regions - including the dorsomedial prefrontal cortex (dmPFC), ventromedial prefrontal cortex (vmPFC), and ventrolateral prefrontal cortex (vlPFC) - is variable, reflecting heterogeneity in clinical presentation. In contrast, limbic and cingulate regions generally demonstrate increased activity across studies, and elevated cerebellar activity has also been reported. Red markers denote regions of increased activity, blue markers indicate reduced activity, and question marks highlight areas where findings remain variable, inconsistent, or insufficiently characterized.

BD has been shown to be linked to state-dependent changes in CBF. For instance, a 320-slice computed tomography study demonstrated increased rCBF in the hippocampus and medial temporal lobe of BD-I patients in the manic state compared to the resting state, with concomitant increases in blood flow velocities of the left middle cerebral artery and right internal carotid artery (Figure 4; Wang et al., 2018). In the depressive state, there was reduced hippocampal and temporal rCBF. This is consistent with structural decreases that are commonly seen in these areas.

Volume decreases in the hippocampus and amygdala are among the most replicable neuroanatomical findings in BD. Curiously, smaller hippocampal or amygdalar volume have also been associated with increased blood oxygen level-dependent (BOLD) signal or rCBF during emotional or rewards processing (Savitz and Drevets, 2011). This function-structure paradox - increased functional activity in a structurally impaired region - may represent a form of compensatory metabolic recruitment, such that these circuits are working harder (at a metabolic level) to maintain processing capacity. This compensatory hyperactivation could over time drive excitotoxicity and neurodegeneration, perhaps contributing to the progressive gray matter volume loss that is sometimes seen in BD (Toma et al., 2018).

There is also evidence from perfusion imaging to support state-dependent fronto-limbic imbalance. SPECT studies often describe limbic hyperperfusion in conjunction with relative prefrontal hypoperfusion in the manic state, with some reversal of this pattern during the depressive state (Estudillo-Guerra et al., 2020). In an early SPECT study, both prefrontal and limbic perfusion was decreased in bipolar depression, which was later recapitulated with fMRI (Ito et al., 1996; Wei et al., 2018). Notably, limbic hyperactivity during the depressive state has also been described (Ketter et al., 2001; McLean et al., 2022), suggesting that emotion salience networks may be dysregulated even when cortical modulatory systems are suppressed.

In addition to reduced prefrontal activity during both mania and depression, there have been some reports of paradoxical cortical hyperactivation, particularly in the manic or mixed states (Agarwal et al., 2008; McLean et al., 2022). This could represent an effort by cortical control systems to inhibit or dampen limbic overdrive. Limbic hyperactivity and concomitant prefrontal dysregulation have been similarly described in the attention dysregulation syndromes of “limbic ADD,” while more global cortical hyperactivation is thought to be present in the “ring of fire” phenotypes, who present with increased emotional reactivity (Arnsten, 2009; McLean et al., 2022). These cortical-limbic imbalances within BD could drive emotional disinhibition, hypervigilance, obsessive thinking, and behavioral rigidity. The simultaneous presence of increased cortical engagement with continued emotional dysregulation may reflect a failure of these compensatory prefrontal mechanisms to fully recalibrate regulation within emotional circuitry.

Task-based neuroimaging further contextualizes how changes in CBF may relate to cognitive performance and mood-state-dependent neural recruitment. BD has been marked by dysregulated temporal, limbic, and prefrontal network recruitment across a range of tasks. One SPECT study investigated CBF during the Verbal Fluency Test (VFT) and found exaggerated perfusion of the temporal pole (TP), a region associated with emotional - language integration (Estudillo-Guerra et al., 2020). Another study found that over-recruitment of the anterior and posterior temporal cortices correlated with poor attention and psychomotor speed scores (Benabarre et al., 2005). This relationship between elevated temporal perfusion and poor task performance may point to inefficient neural engagement during episodes of BD.

In contrast to hyperactivation of temporal regions, several studies have found hypo-perfusion in frontal regulatory areas during cognitive challenges. Estudillo-Guerra et al. (2020) found lower perfusion in the right orbitofrontal cortex (OFC) during the Working Memory Test (WMT), in line with observations of executive dysfunction. The OFC is known to dampen limbic excitability and facilitate response inhibition (Jobson et al., 2021). Impaired OFC recruitment during the working memory task may indicate reduced top-down inhibition of emotional responses. Reduced left parietal perfusion has also been shown to relate to decreased working memory and attentional scores (Benabarre et al., 2005).

These task-based studies have also uncovered mood-state–specific perfusion signatures. Across a variety of tasks - the Immediate Verbal Learning Test (VLT-I), Delayed Verbal Learning Test (VLT-D), Working Memory Test (WMT), Verbal Fluency Test (VFT), and Processing Speed Test (PST) - unique regional patterns of CBF shifts were present. For instance, left TP hyperperfusion was found in both verbal learning and fluency conditions, while hypo-perfusion of the orbitofrontal cortex was seen during the working memory task (Estudillo-Guerra et al., 2020). This pattern is similar to previous reports of hyperactivated amygdala and reduced OFC activity during affective labeling tasks in manic patients (Altshuler et al., 2005), supporting the idea that increased emotional salience during episodes hinders cognitive control systems. There is also evidence suggesting that OFC hypoactivity might be a more consistent feature of BD. Lower OFC perfusion has been reported both during word-generation tasks and at rest (Blumberg et al., 1999; McLean et al., 2022), which may indicate that these prefrontal regulatory deficits extend to non-task conditions. In a more recent meta-analysis, Ahmed et al. (2023) also reported that OFC hypoactivation was most robust in manic states, while bipolar depression may be associated with greater OFC activity during affective labeling tasks, which may indicate greater inhibition of emotional responses.

Additional task-based fMRI studies reinforce the presence of mood-dependent corticolimbic imbalances (Chen et al., 2011). During negative facial emotion processing, manic patients exhibit reduced activation in the left lateral OFC but increased activity in the dorsal ACC, insula, and dlPFC) relative to controls (Hulvershorn et al., 2012). In contrast, euthymic BD and borderline personality disorder groups show heightened amygdala responses to negative stimuli, indicating persistent sensitivity within emotional salience networks.

Spatial attention paradigms provide further support for disrupted cortical regulation. During the Processing Speed Test (PST), decreased ACC perfusion has been observed, consistent with reduced cognitive control during manic states. In contrast, increased perfusion in the temporal pole during emotional and cognitive tasks suggests exaggerated emotional salience processing, whereas diminished activity in regulatory regions such as the OFC and ACC reflects impaired executive control. Supporting this interpretation, increased temporal perfusion has been linked to poorer attention and psychomotor performance, while reduced frontal perfusion correlates with deficits in executive and memory function (Benabarre et al., 2005; Deckersbach et al., 2006).

Positron emission tomography with ^18^F-fluorodeoxyglucose (FDG) has also offered critical insight into alterations in cerebral glucose metabolism, further supporting the presence of state-dependent neurophysiological alterations in the limbic and prefrontal circuitry of BD. Multiple studies have reported regional hyperactivity of regulatory cingulate circuitry in the context of manic states. Blumberg et al. (2000) reported increased metabolism in the left dorsal ACC in a group of manic patients at rest, findings that are echoed by SPECT studies of increased ACC activity in the context of limbic hyperactivation and lateralized thalamic asymmetry during manic states (McLean et al., 2022). Further task-based studies also support a positive correlation between manic symptom severity and dorsal ACC activation, and also implicate decreased metabolism in the right frontal pole as a potential biomarker of deficient regulatory control within prefrontal circuitry (Rubinsztein et al., 2001). Initial PET work also found a similar pattern of increased ventral subgenual cingulate metabolism in the context of mania, which contrasted with decreased activity observed during bipolar and unipolar depression (Drevets et al., 1997).

There may be an important link between cingulate hyperactivity and underlying structural abnormalities. Large-scale multimodal imaging work has also found reduced surface area in the right caudal ACC as a part of a network of local cortical alterations among a sample of bipolar patients with a history of psychosis, highlighting the potential for persistent metabolic overactivation to drive regionally specific cortical remodeling over time (Hibar et al., 2018). Beyond cortical circuits, metabolic imaging has implicated robust subcortical involvement as well. Metabolism of the thalamus, striatum, and right amygdala were found to be elevated while metabolism of the prefrontal and paralimbic cortex was found to be reduced in a sample of treatment-resistant and rapid-cycling bipolar patients (Ketter et al., 2001). Although hyperactivity of the amygdala during emotion processing has been consistently replicated in bipolar depression, the data supporting its persistence in resting-state is more equivocal (Brooks et al., 2009).

In addition to these findings, notable changes in metabolism have been observed in the cerebellum. Resting state PET and SPECT studies have both shown increases in cerebellar metabolism across mood states (Ketter et al., 2001; McLean et al., 2022), highlighting the importance of cerebellar-limbic circuitry in affective modulation. While the precise relationship between cerebellar activity and mood-state switching remains to be defined, these findings highlight that affective instability in BD is likely characterized by abnormalities in distributed cortico-subcortical networks as opposed to focal limbic pathophysiology.

**FIGURE 5:**
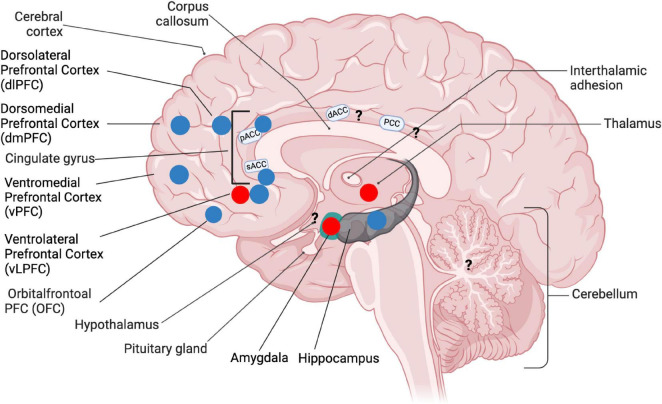
Bipolar depression at rest. This schematic illustrates regional patterns of brain activity associated with bipolar depression under resting-state conditions. The amygdala and thalamus frequently demonstrate increased activity. Activity within the ventrolateral prefrontal cortex (vlPFC) is variable, with reports of both increased and reduced activation across studies. In contrast, the cingulate gyrus, ventromedial prefrontal cortex (vmPFC), orbitofrontal cortex (OFC), dorsomedial prefrontal cortex (dmPFC), and dorsolateral prefrontal cortex (dlPFC) generally exhibit reduced activity. Red markers denote regions of increased activity, blue markers indicate reduced activity, and question marks highlight areas where findings remain variable, inconsistent, or insufficiently characterized with respect to resting-state conditions.

**FIGURE 6:**
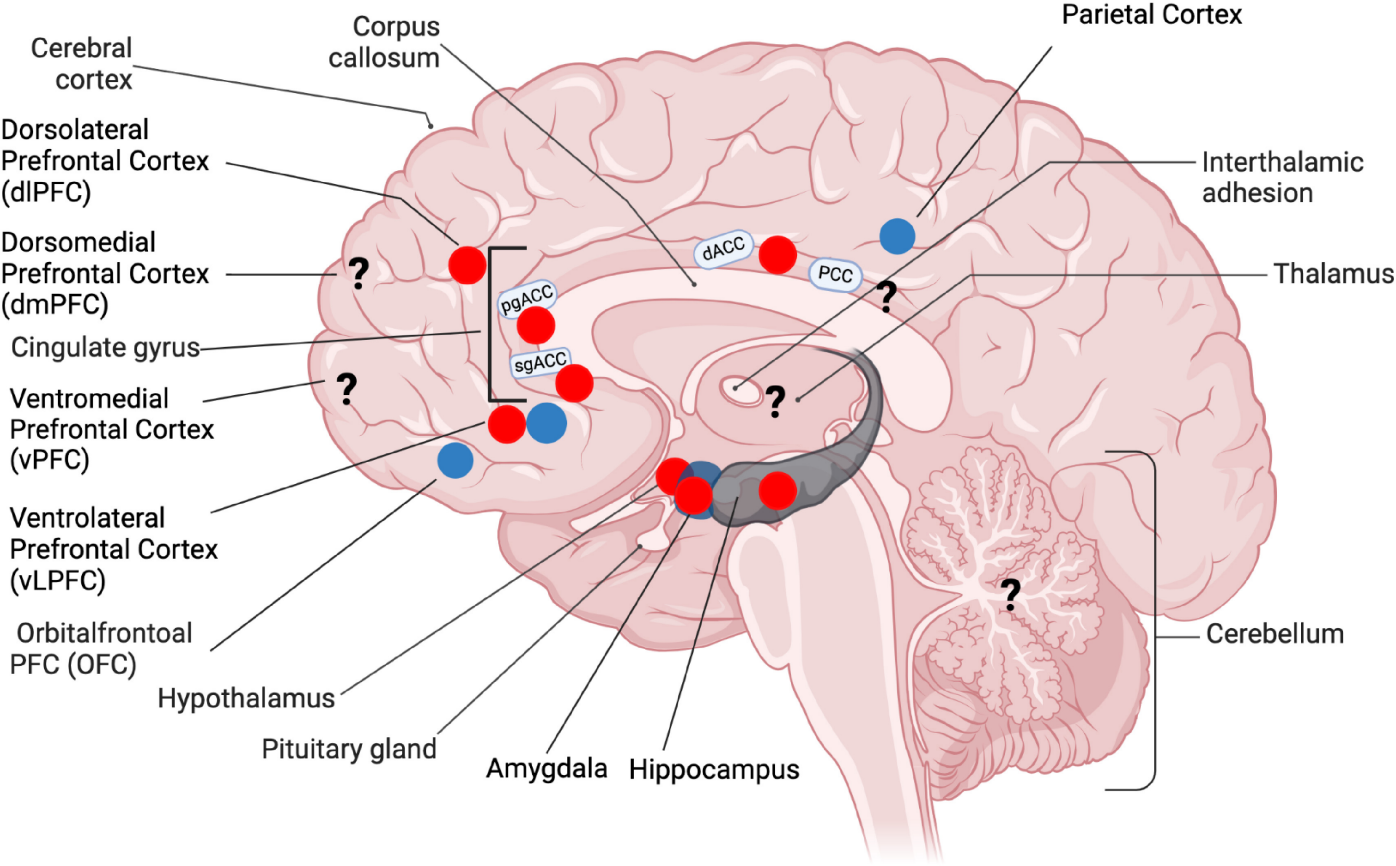
Executive cognition in bipolar mania. This schematic illustrates regional patterns of brain activity observed during executive cognition tasks in bipolar mania. Increased activity is commonly reported in the dorsolateral prefrontal cortex (dlPFC), cingulate gyrus, hypothalamus, amygdala, and hippocampus. Activity within the ventromedial prefrontal cortex (vmPFC) appears variable across studies, reflecting heterogeneity in task conditions and clinical presentation. Red markers denote regions of increased activity, blue markers indicate reduced activity, and question marks highlight areas where findings remain variable, inconsistent, or insufficiently characterized with respect to task-specific activation patterns.

**FIGURE 7:**
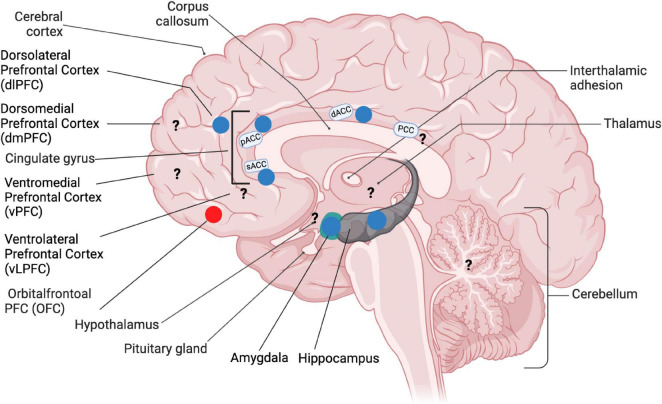
Executive cognition in bipolar depression. This schematic illustrates regional patterns of brain activity observed during executive cognition tasks in bipolar depression. Reduced activity is commonly reported in the dorsolateral prefrontal cortex (dlPFC), cingulate gyrus, hypothalamus, amygdala, and hippocampus. In contrast, the ventrolateral prefrontal cortex (vlPFC) often demonstrates increased activation. Red markers denote regions of increased activity, blue markers indicate reduced activity, and question marks highlight areas where findings remain variable, inconsistent, or insufficiently characterized with respect to task-specific activation patterns.

For a precise visual reference of the brain regions and activation patterns described above, see Figures 2–7.

## A big picture - integrative model

The systems discussed in the previous sections, namely, genetic and epigenetic predisposition, HPA axis dysregulation, mitochondrial and metabolic dysfunction, immune and inflammatory activation, gut-brain dysbiosis, and prefrontal-limbic imbalance, do not, I would argue, independently contribute to the pathophysiology of BD. Rather, the main thesis of this review is that these systems form a hierarchically arranged, bidirectionally entrained feedback mechanism which, by virtue of its extreme instability, results in the kind of cyclic mood switching that is the hallmark of this illness.

The sequence of events that’ve been laid out are the following. Genetically programmed variations in key enzymes and signaling proteins such as COMT, MTHFR, GSK3β, CLOCK, PER3, BMAL1, FKBP5, and many others not discussed in this review, create individual thresholds for stress reactivity, dopamine homeostasis, and clock gene entrainment. An environmental insult, usually a form of early life stres, acting on this predisposition, leads to epigenetic modification, most prominently at the FKBP5 gene locus. The epigenetic change has the downstream effect of glucocorticoid receptor downregulation and impaired HPA axis negative feedback function, resulting in chronic low-level hypercortisolemia. This is, of course, a simplification, as many people with BD may not exhibit overt HPA axis dysfunction in the same way. In the literature, this has been described as a trait-level dysfunction with state-dependent modulation. HPA axis dysregulation in this context should be seen as a disorder of glucocorticoid feedback rather than absolute hypercortisolism.

Chronic cortisol elevation, along with clock gene variation and sleep-wake cycle disruption, creates a state of ongoing bioenergetic stress on the neuron. Neuronal mitochondria are unable to adequately supply energy via oxidative phosphorylation and oxidative metabolism is complemented by aerobic glycolysis and glutaminolysis - particularly in regions of high metabolic demand, such as the ACC. This state of decreased ATP availability leads to a destabilization of the neuron’s ion gradient and increased vulnerability to calcium dysregulation and excitotoxicity. In this model, depression is characterized by hypometabolism and underactivity of the neuron. Mania, by contrast, would be characterized by a period of temporarily increased energy demand, increased catecholaminergic firing, and neuronal overexcitability, in line with the FDG-PET findings I described above.

This metabolic dysfunction, however, is not isolated. Mitochondrial dysfunction *per se* conditionally enhances pro-inflammatory signaling, and inflammatory cytokines - peripheral ones, likely derived from a dysbiotic gut with increased epithelial permeability - can further exacerbate mitochondrial dysfunction and activate microglia. The resultant neuroinflammatory environment is associated with synaptic remodeling, white matter abnormalities, and volume loss in key structures such as the hippocampus over the long term, bridging the gap between acute episode biology and neuroprogression across the life course of BD. In this way, these processes are all tightly linked, and the relationship is reciprocal - prolonged cortisol elevation itself can cause intestinal hyperpermeability via mast cell degranulation and cytokine signaling. In other words, neuroendocrine dysfunction not only drives, but is driven by gut-brain dysbiosis which in turn drives systemic inflammation.

**FIGURE 8:**
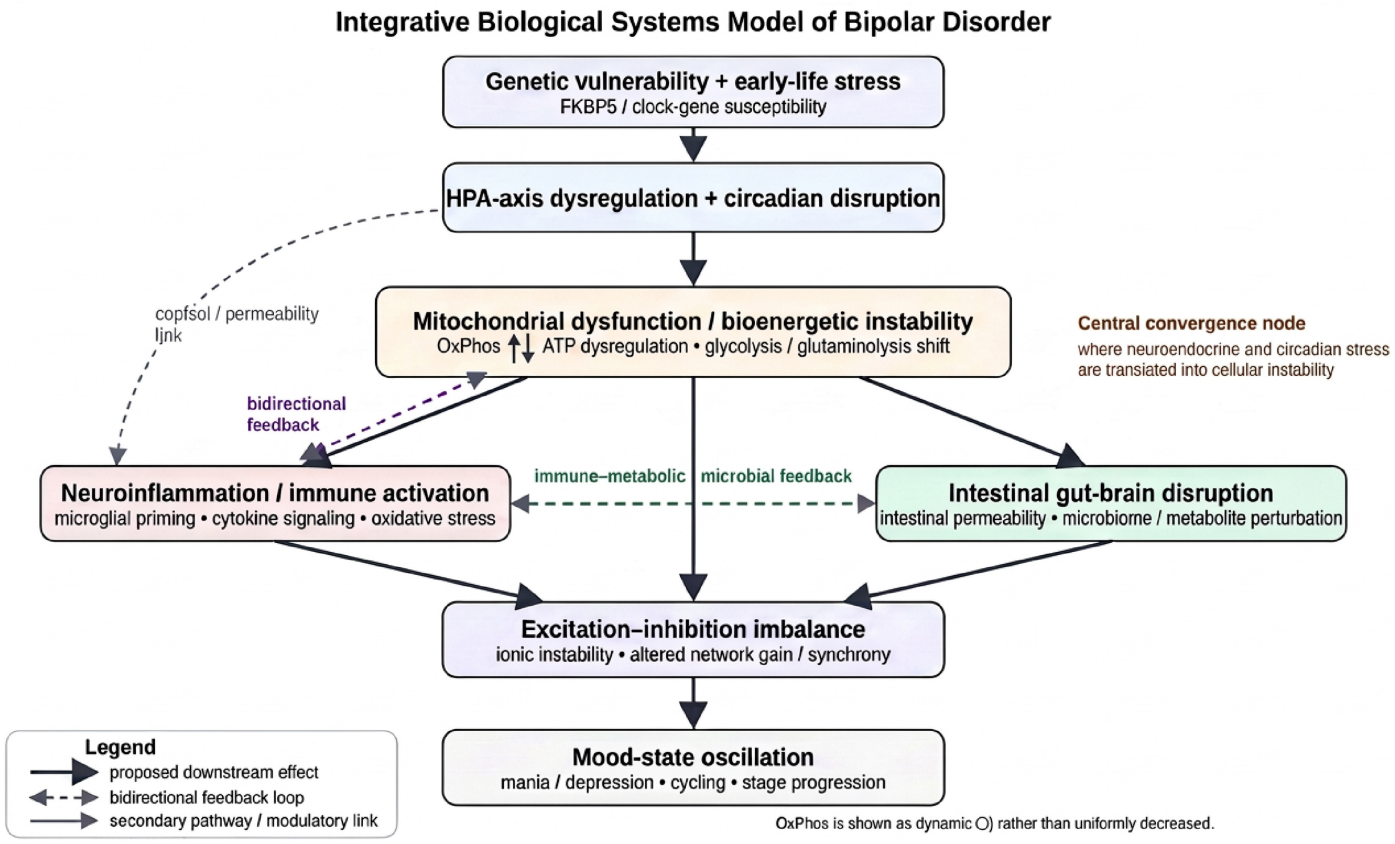
Integrative systems model of bipolar disorder. Genetic and epigenetic vulnerability establishes differential thresholds for stress responsivity and circadian entrainment, which converge on mitochondrial dysfunction as the central integrative node. Solid arrows indicate downstream effects; dashed double-headed arrows indicate bidirectional feedback; faint dashed arrows indicate cross-system links. ↑↓ beside OXPHOS indicates that oxidative phosphorylation is state-dependent, with transient hypermetabolism preceding manic states and hypometabolism characterizing depressive states, rather than being uniformly suppressed. HPA, hypothalamic-pituitary-adrenal; OXPHOS, oxidative phosphorylation; LPS, lipopolysaccharide; GABA, gamma-aminobutyric acid; ACC, anterior cingulate cortex; E/I, excitation-inhibition.

Depression and mania, in this model, are not discrete states but rather the extreme poles of a highly unstable system. Mood state is therefore not the product of a single system going awry, but rather the temporary relative dominance of various metabolic, neuroendocrine, and inflammatory factors - a difference in balance rather than function. This has three key testable implications. First, a panel of inflammatory-metabolic biomarkers at baseline, such as those proposed above (hsCRP, insulin, FDG-PET, HCC, and others) should be able to identify biologically distinct patient subtypes with reliably different illness courses and responses to treatment. Second, the cascade I have outlined here predicts a mood-stabilizing effect of metabolic-inflammation targeted interventions, and this effect size should correlate with baseline mitochondrial and inflammatory dysfunction severity rather than baseline symptom severity alone. Third, and most importantly, I predict that neuroendocrine and metabolic HPA axis activity, mitochondrial function indices, and gut permeability biomarkers tracked longitudinally in parallel with mood state over time will demonstrate a temporally specific relationship - that neuroendocrine and metabolic aberrations come first and mood-state transition second. This set of predictions, I believe, distinguishes this model from simple descriptive multisystem accounts and provides a point of departure for the design of future mechanistically driven research in BD. Figure 8 provides a visual representation of the explanatory model proposed here.

This systems-based framework has implications not only for how BD is treated, but also for how it is diagnosed. One of the most pressing clinical dilemmas is the differential diagnosis of BD from major depressive disorder (MDD), with strong evidence for substantial proportions of BD patients being initially misdiagnosed with MDD, and possibly remaining misdiagnosed for years. As the integrative model places neuroinflammatory signaling, and specifically microglial activation, as the hub mediating BD pathophysiology, this biological substrate itself may provide a diagnostic signal. Zambrano et al. (2026) recently reported a proof-of-concept diagnostic platform in which patient-derived plasma small extracellular vesicles (sEVs) were applied to cultured microglial cells, which then underwent disease-specific morphological changes that were captured by fluorescence microscopy and classified using a DenseNet121 convolutional neural network. This deep learning pipeline correctly classified 44 of 45 subjects across BD, MDD, and healthy control groups with subject-level accuracy of 98.67%. This approach is notable in the present context because it does not treat neuroinflammation as a downstream consequence to be measured after diagnosis, but as a biologically embedded signal that encodes diagnostic information accessible through peripheral blood. The microglial morphological response to sEVs effectively translates the molecular disease state into a measurable cellular phenotype - precisely the kind of systems-level, immune-metabolic readout that the integrative model predicts should differentiate biological subtypes. While the sample size is still small and replication in larger, medication-stratified cohorts is needed, this work highlights how a mechanistically grounded understanding of BD as a neuroinflammatory disorder may generate novel diagnostic tools that move beyond symptom-based classification toward biologically precise identification of the condition.

## Implications for clinical management and modulative interventions for bipolar disorder

Although the primary focus of this review is an integrative synthesis of the existing literature within the domain BD research, the condition itself is ultimately expressed as a clinical syndrome requiring therapeutic intervention. Notably, conventional treatment strategies were largely developed within symptom-based frameworks rather than through integrative biological models. However, many first-line interventions intersect - directly or indirectly - with the biological domains discussed above. Examining these treatments through an integrative lens clarifies both their mechanistic relevance and their limitations.

### Lithium

Consistent with its profile as a first-line treatment, lithium is the most effective mood stabilizer to date for prevention of relapse and suicide risk in BD (Volkmann et al., 2020). A number of intracellular and extracellular signaling pathways have been proposed to mediate lithium’s therapeutic effects. One of the key mechanisms of action of lithium is inhibition of glycogen synthase kinase-3β (GSK-3β), a signaling kinase involved in regulation of neuronal excitability, synaptic plasticity, and mood. GSK-3β inhibition is thought to occur through direct and indirect mechanisms: Lithium is able to occupy a magnesium-binding site on the kinase, and may also enhance the inhibitory phosphorylation of GSK-3β (Bortolozzi et al., 2024). Overactivation of GSK-3β has been shown to disrupt presynaptic neurotransmitter release by hyperphosphorylation of proteins involved in vesicle fusion machinery. For instance, phosphorylation of synaptic proteins that are part of the SNARE complex, such as syntaxin or synapsin-1, impairs vesicle docking and fusion at the presynaptic membrane (Sun et al., 2025). GSK-3β also appears to modulate N-type VDCCs, such as the CACNA1 subunits: phosphorylation of these channels decreases calcium influx and thus calcium-dependent neurotransmitter release. In addition to modulating neurotransmitter release, GSK-3β also has downstream effects on other signaling proteins and gene expression that are thought to underlie some of lithium’s mood stabilizing effects.

Genetic findings in BD also point to involvement of these signaling pathways. For example, the gene encoding the scaffolding protein ankyrin-G (encoded by ANK3) has been repeatedly implicated as a risk locus in GWA studies of BD. Ankyrin-G is important for clustering and stabilizing voltage-gated sodium and potassium channels at the axon initial segment (AIS), a region critical for action potential initiation and regulation of neuronal excitability. In an Ank3 variant mouse model, lithium-induced inhibition of GSK-3β rescued inhibitory neuronal function and normalized circuit excitability (Caballero-Florán et al., 2025).

Lithium also directly alters neuronal electrophysiology through modulation of ion transport and channel conductance. In initial electrophysiological studies, lithium was shown to influence sodium and potassium ion fluxes, leading to prolonged neuronal refractory periods and a decrease in aberrant firing (Ploeger, 1974). Lithium has also been shown to enhance GABAergic inhibitory transmission and partially restore the structure of the AIS, leading to a decrease in hyperexcitability of pyramidal neurons. Functionally, ankyrin-G directly interacts with GABA-receptor-associated protein (GABARAP), a protein that helps maintain the integrity of inhibitory synapses. Mutations in ANK3, including the p.W1989R variant, lead to disruption of the ANK3-GABARAP interaction, resulting in increased endocytosis of GABA receptors from the surface of postsynaptic pyramidal neurons and a reduction in the number of inhibitory synapses (Nelson et al., 2020; Tseng et al., 2015).

Although lithium has also been shown to increase gephyrin clustering at inhibitory synapses through GSK-3β inhibition (Tyagarajan et al., 2011), lithium does not appear to reverse the loss of inhibitory synapses or surface GABA-A receptor density in the ANK3 p.W1989R model. Rather, lithium appears to compensate for this deficit at the level of synaptic function. Ank3 mutant mice have increased action potential firing rates in pyramidal neurons, a phenotype which is normalized by chronic lithium treatment to wild-type levels. Importantly, lithium restores the frequency (but not the amplitude) of spontaneous inhibitory postsynaptic currents (sIPSCs), indicating that lithium increases inhibitory drive primarily by increasing the presynaptic release of GABA from cortical interneurons. The resulting increase in inhibitory signaling helps suppress hyperexcitability in pyramidal neurons and rebalance E/I homeostasis in cortical circuits.

Notably, these mechanistic effects have been recapitulated in clinical outcomes observed in patients on chronic lithium treatment. While its therapeutic effects appear to be more robust for mania than for bipolar depression, multiple meta-analyses of long-term, randomized, placebo-controlled trials have shown that lithium treatment significantly reduces risk of recurrent mood episodes compared with placebo (Geddes et al., 2004; Severus et al., 2014; Miura et al., 2014; Vieta et al., 2011). Evidence for lithium’s efficacy in bipolar depression has been more variable, with some studies finding no significant differences between lithium and placebo or active comparators for depressive episodes (Young et al., 2010; Amsterdam and Shults, 2008). In many of these trials, serum lithium concentrations were relatively low, which may partly account for the lack of antidepressant effect. Taken together, pooled evidence across trials and clinical studies points to a large relapse-preventive effect. A Cochrane review found that the 1-year risk of relapse was approximately 36% in the lithium group versus 61% in the placebo group, corresponding to an absolute risk reduction of 25% and a number needed to treat (NNT) of 4 (Burgess et al., 2001). Observational studies performed under real-world treatment conditions have further supported lithium’s maintenance efficacy. In large registry-based analyses, lithium monotherapy was found to be superior to other mood stabilizer monotherapies in terms of relapse prevention and treatment failure in BD (Kessing et al., 2018; Hayes et al., 2016).

Treatment of bipolar episodes with mixed features remains a particular clinical challenge, in part because relatively few RCTs have specifically examined this patient subgroup. Consequently, the evidence for lithium’s efficacy in mood episodes with mixed features is mixed and less well understood (Betzler et al., 2017; Chakrabarty et al., 2020). Some studies have suggested that lithium may be less effective than other treatments for manic episodes accompanied by co-occurring depressive symptoms. For instance, Swann et al. (1997) reported that lithium had lower efficacy than valproate in mania with mixed features. Although lithium is a first-line maintenance therapy for BD, treatment decisions should be individualized and the clinical benefits carefully weighed against potential adverse effects. These include hypothyroidism (Kibirige et al., 2013; Joffe, 2002), hyperparathyroidism, cardiac conduction changes (such as T-wave inversions), and nephrotoxicity (Volkmann et al., 2020).

### Antipsychotics

Antipsychotics used to treat BD are often categorized into three broad classes: first-generation (typical), second-generation (atypical), and, more recently, third-generation agents. The clinical importance of lithium in certain treatment contexts has been relatively diminished in recent years due to the rapid increase in availability of antipsychotics approved for use in BD (particularly for the treatment of acute mania). Contributing to the widespread use of second-generation antipsychotics (SGAs) in the treatment of acute mood episodes are their relatively rapid onset of action, their sedating effects (useful in controlling agitation), and a broader therapeutic window than that of lithium. Haloperidol (Haldol) is a first-generation antipsychotics (FGA) with a long history of strong antimanic efficacy, even in some studies producing greater short-term symptom reduction than lithium. Haloperidol’s primary mechanism of action appears to be dopamine D2 receptor antagonism (this is the case for all antipsychotics), which results in decreased dopaminergic signaling (Seeman, 2021). There is also emerging evidence that haloperidol has allosteric inhibitory activity at the 5-HT3 receptor, which may be relevant to its overall neuromodulatory activity (Park et al., 2025).

Haloperidol has become less frequently used in recent years due to safety concerns. These include a relatively high propensity for extrapyramidal symptoms and movement disorders such as tardive dyskinesia and akathisia (relatively common with all high-potency first-generation antipsychotics), QT-interval prolongation, and increased mortality in elderly patients with dementia when used (Essali et al., 2019; Ehler and Petzold, 2025). The possibility of neurotoxicity with haloperidol (as well as other first-generation antipsychotics) has also led to a more conservative approach to the drug in recent years relative to the use of newer antipsychotic agents (Nasrallah and Chen, 2017).

Second-generation antipsychotics (SGAs) remain first-line in the treatment of acute mania due to their strong antimanic efficacy and typically improved tolerability profile versus first-generation agents. The majority of SGAs are approved for manic or mixed episodes in adult and pediatric populations, and are frequently used either as monotherapy or in combination with mood stabilizers such as lithium or valproate.

Olanzapine has reliably demonstrated strong antimanic effects at doses of 10–20 mg/day and is commonly considered one of the most efficacious SGAs for acute mania. Intramuscular formulations may be particularly helpful for controlling agitation, and combination therapy with lithium or valproate may further augment treatment response. Quetiapine is similarly effective in manic episodes at doses of approximately 400–800 mg/day, and is comparable to lithium in efficacy in some studies. As with olanzapine, combination with mood stabilizers may be helpful to improve outcomes relative to monotherapy.

Risperidone has consistently demonstrated robust antimanic efficacy at doses of 1–6 mg/day, and is frequently ranked among the more efficacious SGAs in acute mania (Sajatovic et al., 2006). Both its efficacy and safety profile are comparable to halidol (Yildiz et al., 2011). Combination therapy with lithium or valproate has been found to further augment its efficacy. Asenapine and ziprasidone have also shown efficacy in manic episodes and are approved for this indication in many regulatory jurisdictions. Asenapine is frequently recommended in treatment guidelines for acute mania.

Clozapine is not formally approved for the treatment of mania and is generally considered an off-label option for this indication, given the lack of double-blind randomized controlled trials to date. However, clinical experience and case studies do suggest that clozapine may be effective in treatment-resistant manic states, and systematic reviews have reported antimanic efficacy similar to that of other antipsychotics (Delgado et al., 2020). In the most recent meta-analysis and systematic review the most commonly reported adverse effects were sedation (49.6%), followed by constipation (31.8%) and tachycardia (23.2%) (Delgado et al., 2020). Interestingly, given the current pharmacodynamic theories regarding the action of Clozapine, its mechanism of action may reach far beyond actions on dopamine receptors, as its receptor binding affinity is considerably smaller than its counterparts, in-spite of reports of comparable efficacy (Hengartner and Moncrieff, 2018).

Third-generation antipsychotics have expanded the pharmacological landscape for BD and are increasingly used across manic, depressive, and maintenance phases of the illness. These agents - which include aripiprazole, cariprazine, lurasidone, lumateperone, and ziprasidone - differ mechanistically from previous antipsychotic classes in that they act as partial dopamine receptor agonists while also modulating serotonergic signaling. This unique pharmacological profile may stabilize dopaminergic transmission, rather than fully suppressing it, which may in part account for their clinical utility and tolerability.

In the management of acute mania, several third-generation antipsychotics have demonstrated therapeutic benefit. Aripiprazole, typically given at doses of approximately 15–30 mg/day, has demonstrated antimanic efficacy comparable to lithium in some trials, and intramuscular formulations may be useful for controlling agitation during acute episodes. Combination of aripiprazole with mood stabilizers such as lithium or valproate can further improve outcomes. Cariprazine, a dopamine D3-preferring partial agonist, has also demonstrated efficacy in the treatment of acute manic and mixed episodes. Randomized placebo-controlled studies have found that doses in the range of 3–12 mg/day produce significant reductions in manic symptoms.

The treatment of bipolar depression is more limited, but several agents in this class have demonstrated benefit. Lurasidone, typically given at doses between 20 and 120 mg/day, has been shown to produce clinically meaningful reductions in depressive symptoms when used as monotherapy or in combination with lithium or valproate. Meta-analyses and systematic reviews have confirmed these antidepressant effects. Cariprazine was recently FDA-approved for bipolar depression based on randomized controlled trials showing efficacy in doses of approximately 1.5–3 mg/day. Similarly, lumateperone, which was studied at 42 mg/day in phase III trials, demonstrated significant improvements in depressive symptoms in patients with bipolar I or bipolar II disorder and maintained a favorable tolerability profile. Although aripiprazole is less consistently effective as monotherapy for bipolar depression, lower doses (approximately 5–10 mg/day) are sometimes used in clinical practice as augmentation therapy in cases where antidepressant response is partial or absent.

Some third-generation antipsychotics have also demonstrated efficacy in maintenance treatment. Long-term studies have shown that aripiprazole can prevent recurrence of manic episodes when used as monotherapy, although its effect on preventing depressive relapse appears more limited. Augmentation of lithium or valproate with aripiprazole has shown stronger prophylactic benefits. Long-term treatment with lurasidone has also been associated with sustained improvements in depressive symptoms and acceptable safety profiles when used as monotherapy or in combination with mood stabilizers. Finally, although ziprasidone has demonstrated efficacy in the treatment of BD, some reports suggest that it may on occasion be associated with emergence of manic symptoms, highlighting the complex pharmacodynamic effects of dopamine-modulating agents (Baldassano et al., 2003).

### Antidepressants (SSRIs, SNRIs & tricyclic antidepressants)

The role of antidepressants in bipolar depression is one of the most controversial topics in psychopharmacology. Although antidepressants are a mainstay in the treatment of major depressive disorder, data supporting antidepressant efficacy in bipolar depression is relatively limited, but antidepressant use is nevertheless common in clinical practice. Estimates vary, but in a systematic review and meta-analysis, Pardossi et al. (2025) found that the pooled proportion of individuals with BD who receive antidepressants at any time during treatment was 54% (95% CI, 50–57.6). As depressive episodes are often the most frequent or severe in the clinical course of BD, antidepressant use is often considered, but their use has also historically been approached with great caution due to a number of theories and reports related to treatment emergent mania, cycle acceleration, and general mood destabilization (Gitlin, 2018). Treatment guidelines therefore typically discourage antidepressant monotherapy in BD and, instead, suggest that when antidepressants are indicated, they are added on to other mood stabilizing treatments or adjunctive antimanic agents (Gottlieb and Young, 2024; Pacchiarotti et al., 2013; Yatham et al., 2018).

This basic pharmacologic rationale is in part derived from a popular biological model of depression that hypothesizes a fundamental serotonin deficiency. However, the model of depression as resulting from a simple deficiency in serotonin has increasingly been called into question in recent years. A large umbrella review, published in Molecular Psychiatry, analyzed a number of different lines of biological evidence and reported that there was no consistent evidence to suggest that depression is associated with a decreased serotonin function or concentration (Moncrieff et al., 2023). The biological evidence specifically included studies of serotonin metabolites, receptor binding, transporter availability, tryptophan depletion experiments, and genetic associations, among others. The umbrella review concluded that there was no consistent evidence of decreased serotonin activity, but, rather, the effects of antidepressants may instead be the result of more complex, downstream effects, rather than through direct reversal of a monoamine deficiency (Bottaccioli et al., 2025). Although the umbrella review did not directly address bipolar depression, it does still suggest caution in viewing depression (regardless of subtype) as simply resulting from serotonin deficiency, as well as caution in extrapolating our general understanding of depression in unipolar depression to bipolar depression. In the case of BD, this uncertainty is particularly pertinent as it is well known that antidepressants, particularly when used without concomitant mood stabilization, precipitate mood switching, rapid cycling, or manic activation in vulnerable individuals (El-Mallakh et al., 2015).

Nonetheless, some individuals do report clinically meaningful improvements in their depressive symptoms when using antidepressants in conjunction with a mood stabilizer (Pardossi et al., 2025; Jefsen et al., 2023). Meta-analytic data suggest that, while they produce only small to moderate improvements in bipolar depression, antidepressants may have a beneficial effect when used as adjunctive therapy to mood stabilizers; however, the overall quality of the evidence is moderate and the response is not uniform across patients (Oliva et al., 2025). Accordingly, treatment should be individualized to the patient’s past history, current symptom profile, and risk for destabilization.

Other antidepressant agents that are also germane to the treatment of BD, particularly TCAs and serotonin-norepinephrine reuptake inhibitors (SNRIs, specifically Venlafaxine) appear more strongly tied to manic switching or cycle acceleration, when compared to SSRIs, likely due to their additional monoamine stimulation (Kurita, 2016). For this reason, newer generation antidepressants are typically favored, and if these agents are prescribed, they are used with careful patient selection and close monitoring in individuals with BD (Vázquez and Baldessarini, 2025).

### Anti-convulsants

Some common anticonvulsants such as lamotrigine, valproate (divalproex), and carbamazepine are also used in BD. Lamotrigine is the most commonly used anticonvulsant mood stabilizer in the treatment and prevention of bipolar depression, in people with bipolar I disorder, with evidence also for benefit in bipolar II disorder (Prabhavalkar et al., 2015). In the treatment of epilepsy, lamotrigine’s major antiepileptic actions are via voltage-gated sodium and calcium channel blockade to decrease release of the excitatory neurotransmitter glutamate (Costa and Vale, 2023). This likely underlies at least some of lamotrigine’s mood-stabilizing effects, although the exact mechanisms underlying its efficacy in bipolar depression are not well defined. Experimental data have shown lamotrigine to also modulate serotonergic neurotransmission, including agonism of 5-HT1A receptors, which is thought to be linked to upregulation of adenylate cyclase signaling in the cortex (Vinod and Subhash, 2002). Of anticonvulsants used in BD, lamotrigine has the most evidence for efficacy in depressive episodes of the illness, and has a lower risk of manic switching relative to conventional antidepressants, though more limited evidence for acute mania (Prabhavalkar et al., 2015).

### Psychotherapeutic and behavioral interventions

Several psychotherapies are commonly employed in clinical practice as adjuncts to pharmacotherapy in BD. These interventions are typically behavioral in nature, delivered as multi-session individual or group therapies designed to stabilize behavioral patterns and improve stress regulation, thereby decreasing risk for relapse. By these mechanisms, behavioral therapies may indirectly modulate biological systems that are dysregulated in BD, such as circadian rhythms, stress reactivity, or neural systems supporting emotion regulation.

Psychoeducation is often implemented early in treatment and typically takes the form of structured individual or group sessions across several weeks. The primary goal is to help individuals with BD gain a clear understanding of the illness and a rationale for treatment engagement and adherence. Sessions usually include education on how to recognize symptoms and relapse triggers, manage stressors, and adhere to pharmacotherapy. Clinicians also provide information on BD course, common prodromal symptoms of mood episodes, and behavioral strategies to maintain stability. Patients are encouraged to track mood, sleep, and daily activity patterns between sessions, to identify early warning signs and enable both clinician and patient to intervene before full mood episodes occur. In this way, psychoeducation in clinical practice prioritizes illness awareness, treatment adherence, and early relapse detection, which have all been associated with better long-term outcomes (Bahredar et al., 2014). Group psychoeducation has been demonstrated as a particularly effective adjunct to pharmacotherapy, with some studies showing that group education can more than double time to recurrence (Novick and Swartz, 2019).

Cognitive behavioral therapy for BD is generally delivered in structured weekly sessions across several months, either individually or in groups. The approach is based on the idea that thoughts, emotions, and behaviors interact to influence stability of mood, and that changing maladaptive patterns of thought can disrupt recurrent cycles that precipitate mood episodes. Patients are guided to identify unhelpful or distorted patterns of thinking - such as rumination, catastrophic thinking, or goal-directed behavior that is too rigid or intense - and to use cognitive and behavioral strategies to regulate mood, sleep, and daily activity patterns. Common techniques include thought records, mood diaries, activity scheduling, and structured behavioral plans, with home practice exercises reinforcing strategies between sessions (Özdel et al., 2021).

Clinical trials and meta-analyses suggest that CBT is effective at decreasing risk for relapse, improving depressive symptoms, and enhancing psychosocial functioning in BD, particularly as an adjunct to pharmacotherapy (Novick and Swartz, 2019). There is also evidence that CBT may improve time to recurrence of mood episodes and treatment adherence, though these effects may be stronger for depressive symptoms than for acute mania. Family-focused therapy (FFT) is a structured psychotherapy developed to decrease risk for relapse in BD by improving the interpersonal environment surrounding the patient. The intervention is usually delivered through joint sessions involving the patient and one or more family members across several months. Treatment generally occurs in three phases: psychoeducation, communication training, and problem-solving skills development. During psychoeducation, clinicians review the course of BD, the stress–vulnerability model, and the importance of medication adherence. Subsequent sessions target family communication skills through guided interaction exercises and role-play, followed by training in collaborative problem solving to address interpersonal stressors. Because high levels of criticism, hostility, or emotional overinvolvement within families - known collectively as high expressed emotion - have been linked to relapse in BD, FFT is designed to attenuate these interaction patterns and to strengthen supportive family dynamics (Miklowitz and Chung, 2016). RCTs and meta-analyses have demonstrated that FFT can decrease relapse rates, lengthen time to recurrence, improve medication adherence, and reduce depressive symptom burden, especially as an adjunct to pharmacotherapy (Novick and Swartz, 2019). By targeting interpersonal stress in the patient’s immediate environment, FFT represents an important behavioral intervention for long-term stabilization in BD.

Interpersonal and social rhythm therapy (IPSRT) is an intervention developed specifically to target circadian instability, a well-established vulnerability in BD. In clinical practice, patients and therapists use structured tracking tools to map daily routines - such as timing of sleep and waking, meals, social activity, work or school schedule - to identify circadian fluctuations. The focus is on gradually regularizing daily routines so that sleep and daily activities occur at consistent times each day. Interpersonal stressors that may disrupt these rhythms are also addressed in therapy. By helping to regularize daily rhythms, IPSRT may stabilize underlying circadian processes that support sleep, hormonal rhythms, and mood regulation (Steardo et al., 2020).

In this way, these interventions illustrate that behavioral therapies can have the effect of indirect biological modulators in BD. By improving treatment adherence, reducing psychosocial stress, strengthening cognitive control mechanisms, and regularizing circadian rhythms, behavioral therapies target physiological systems that contribute to mood stability and relapse prevention.

## Anti-inflammatory and metabolic adjunctive strategies

Dietary patterns have recently emerged as possible factors influencing brain health and psychiatric outcomes. Diets high in processed and refined foods typical of Western dietary patterns have been linked to brain structural and functional changes, including decreased hippocampal volume and impaired neurogenesis (Jacka et al., 2015; Kishi et al., 2015; Liu et al., 2015). Although the role of nutrition as a central determinant of physical health has long been recognized, its importance for mental health has traditionally been given little consideration. However, increasing evidence from both epidemiological and clinical studies indicates that overall diet quality may be associated with risk and prognosis of psychiatric illness (Lassale et al., 2019). In line with these observations, some recently updated clinical guidelines for mood disorders have begun to include nutrition as an important aspect of treatment (Marx et al., 2017; Platzer et al., 2020).

In addition to its psychiatric symptoms, BD is associated with significantly increased all-cause mortality, due in part to cardiovascular and metabolic comorbidities and also suicide (Dickerson et al., 2017). In light of geographic variability in the prevalence and incidence of BD, increased attention has been placed on environmental and lifestyle risk factors for the disorder, including diet. These epidemiological observations provide a basis for further investigation of nutrition as a potential modifiable risk factor for BD. Therapeutic strategies that ameliorate or circumvent metabolic perturbations therefore have the potential to be of benefit. Given the high comorbidity of metabolic abnormalities in BD, these strategies could also provide benefits to overall physical health. However, metabolic- or inflammation-stratified approaches to these issues are not yet well-developed, and opportunities to tailor such strategies to individual patient needs are not yet available. With these limitations in mind, some new adjunctive treatments are being developed that target metabolic and inflammatory pathways involved in BD pathophysiology.

### Diet & supplementation

Of the dietary interventions that have been examined in the context of BD, the ketogenic diet (KD) has been the most well studied. To date, there are no randomized controlled trials of the KD or of a ketogenic diet in the context of BD, but a number of clinical and pilot studies have demonstrated promising results. The hallmark of the KD is the sustained production of ketone bodies, which are small molecules generated from fatty acid oxidation in the liver and used as an alternative fuel source for the central nervous system and periphery (Napoleão et al., 2021; Tahreem et al., 2022). As a dietary regimen, the KD is defined by the consumption of a high-fat (roughly 65%–80% of calories), moderate protein (20%–25%), and very low carbohydrate (<5%–10%) diet. As a result, the KD places the body in a metabolic state similar to aspects of fasting. The KD was first introduced in the 1920s as a dietary treatment for epilepsy, and since then has been evaluated for many other neurological and metabolic conditions.

The literature evaluating ketogenic diets in the context of bipolar disorder is small but growing. In a recent pilot study, euthymic patients with BD were able to adhere to a modified KD for an average of 6–8 weeks, demonstrating successful induction of nutritional ketosis, high treatment adherence, and mild adverse effects (Needham et al., 2023). In several case reports, extended periods of mood stability have also been described in patients with bipolar II disorder who remained in ketosis for many years. In some cases, the magnitude of these improvements was greater than previously observed with other treatment modalities and even involved decreases in the use of psychiatric medications (Phelps et al., 2013; Chmiel, 2022).

Other observational and clinical studies have provided additional preliminary support for KD and similar dietary approaches in BD. In a retrospective review of a hospitalized cohort of patients with bipolar II disorder, implementation of a strict KD (consumption of less than 20 g of carbohydrates per day) was associated with improvements in a variety of metabolic parameters (body weight, blood pressure, triglycerides, LDL cholesterol) as well as decreases in depressive and psychotic symptomatology (Danan et al., 2022; Needham et al., 2023). In a large online survey of several hundred people with BD, survey participants who reported adherence to ketogenic-style dietary patterns also reported more sustained mood stability when compared to other dietary groups and described improvements in energy, cognition, and weight management (Campbell and Campbell, 2019b). In a more recent short-term clinical survey, individuals with BD and metabolic abnormalities demonstrated significant improvement in overall clinical status when the KD was maintained (Sethi et al., 2024). Although these data are encouraging, there is a clear need for additional controlled trials to better assess the efficacy of KD and the populations that may derive the greatest benefit.

There are several potential mechanistic reasons why nutritional ketosis may be relevant to BD. Ketone bodies play multiple roles in cellular metabolism and bioenergetic function. For example, β-hydroxybutyrate (BHB), the predominant circulating ketone body, can serve as an ATP substrate when PDH is compromised - a consideration with particular relevance given the evidence of impaired PDH in BD (Campbell and Campbell, 2019a). Ketone oxidation also results in the generation of intermediates that feed into mitochondrial oxidative phosphorylation, including succinate which donates electrons to the electron transport chain (ETC) at Complex II (Hwang et al., 2022).

Additionally, oxidation of ketone bodies can support greater ATP production per unit of oxygen consumption when compared to glucose or fatty acid substrates, which may decrease the energetic burden of maintaining neuronal membrane potentials (Norwitz et al., 2019). This effect may be achieved with a lower generation of ROS that is typically observed in conditions of dysfunctional mitochondrial respiration and may even mitigate oxidative stress under conditions of elevated ROS, such as traumatic brain injury models (Greco et al., 2016). Experimental studies have further suggested that chronic exposure to ketone bodies can increase mitochondrial efficiency, possibly by increasing mitochondrial DNA copy number and promoting more stable assembly of respiratory chain complexes, including Complex I (Frey et al., 2017). In addition to their role as metabolic fuels, ketone bodies are also signaling molecules with important roles in mitochondrial biogenesis, including activation of pathways involving PGC-1α, SIRT1, and AMPK (Chen et al., 2025; Gómora-García et al., 2023).

Other dietary patterns, such as the Mediterranean diet and certain well-planned vegetarian or vegan dietary patterns, have also been examined as a potential dietary approach to mood disorders, but have been less well studied specifically in BD. Like the KD, these dietary patterns are generally characterized by greater intake of fruits, vegetables, legumes, whole grains, nuts, and unsaturated fats, while limiting highly processed foods and refined sugars. Evidence from observational and interventional studies suggests that these dietary patterns may promote mental health through a number of potential pathways, including reductions in systemic inflammation, metabolic regulation, and increased micronutrient intake (Akerele et al., 2025; Marano et al., 2025). It is important to note, however, that research suggests that diet quality overall - as opposed to adherence to a specific diet label - is likely a more important consideration in mental health. In particular, dietary patterns that emphasize nutrient density, metabolic stability, and limited processed food intake appear to confer the greatest benefit, regardless of the name or dietary framework.

Interventions targeting the gut microbial community are beginning to emerge as a potential treatment strategy in BD. In a randomized clinical trial, recent manic patients receiving probiotic supplementation with *Lactobacillus rhamnosus* GG and *Bifidobacterium animalis* Bb-12 had significantly fewer rehospitalizations and overall decreased length of hospital stay (Dickerson et al., 2018). Other studies have also found improvements in cognitive performance, attentional function, and manic symptom severity following supplementation with probiotic blends of *Lactobacillus* and *Bifidobacterium* (Reininghaus et al., 2020). Research has also demonstrated that probiotic interventions may reduce rates of rehospitalization while producing meaningful improvements in both depressive symptoms and cognitive functioning in individuals diagnosed with BD (Obi-Azuike et al., 2023). While results have been heterogeneous, likely due to strain-specific effects, differences in treatment duration and other methodological factors, as well as overlapping medication use, these data suggest that modulation of the gut microbial community can impact clinical outcomes in BD. Observations in other contexts also highlight the behavioral effects of microbial disruption. Acute manic episodes have been associated with the use of antibiotics, indicating that abrupt perturbations to the microbial community can disrupt neuroimmune signaling pathways relevant to mood (Yolken et al., 2016).

Nutritional deficiencies or insufficiencies may also exacerbate the metabolic and neurobiological disturbances relevant to BD. Vitamin D is another important nutrient that is involved in neuroimmune signaling, calcium homeostasis, and regulation of gene expression in neural tissue. Suboptimal vitamin D status is associated with greater inflammatory activity and mood dysregulation (Zhou et al., 2024; Cereda et al., 2021), suggesting that insufficient vitamin D may be an important contributing factor to biological vulnerabilities in BD. Therefore, screening for vitamin D insufficiency and supplementation to correct vitamin D deficiency may be an important and clinically relevant treatment consideration in the management of mood symptoms (Cuomo et al., 2019; Habib et al., 2023; İmre et al., 2023). In the context of vitamin D supplementation, other fat-soluble vitamins that are metabolically interrelated, such as vitamins A and K2, may also be important to consider, as these vitamins are also involved in processes of calcium regulation, immune signaling, and metabolic homeostasis (Chatterjee et al., 2023; Khandelwal et al., 2025; Doost et al., 2024).

Micronutrient availability is crucial to maintain mitochondrial energy production, neurotransmitter synthesis and recycling, antioxidant function, and immune system regulation (Han et al., 2025). For example, several B vitamins including thiamine (B1), riboflavin (B2), and niacin (B3), are key cofactors for enzymes in the Krebs cycle and ETC, while vitamin B6 and folate are involved in neurotransmitter synthesis and recycling (serotonin, dopamine, norepinephrine), as well as the conversion of homocysteine to methionine (Han et al., 2025; Wilson et al., 2019). The use of B vitamin supplementation in psychiatry has a long history. Abraham Hoffer first proposed the use of niacin for the treatment of schizophrenia, an approach that has received periodic clinical interest since then (Rudzki et al., 2021; Jonsson, 2018). More recently, vitamins B1 and B6 have shown promise as an adjunctive treatment in BD-I, with improvements in sleep quality and reductions in mania-related symptoms observed with co-supplementation alongside lithium (Zandifar et al., 2024).

Other nutrients, such as magnesium, zinc, and omega-3 fatty acids, have also garnered interest as potential adjunctive treatment strategies for BD due to their anti-inflammatory, neuroprotective, and metabolic regulatory effects (Li et al., 2025; Marano et al., 2026). Although the evidence is still early, these nutrients are thought to modulate the pathways involved in synaptic signaling, mitochondrial function, and immune regulation and may represent promising future targets for the treatment of BD.

## Translational implications of a systems-based model

A systems-based framework makes stratification actionable by linking measurable biological signatures to intervention classes, rather than applying uniform treatment approaches across a biologically heterogeneous population.

Prominent metabolic dysfunction - including impaired energy utilization or mitochondrial inefficiency, characterized by reduced oxidative phosphorylation and increased reliance on glycolysis, and clinically inferred from findings such as elevated lactate on magnetic resonance spectroscopy, altered glucose uptake on FDG-PET, or the presence of metabolic comorbidities such as insulin resistance - represents a candidate profile for interventions that directly support cellular metabolism, such as ketogenic or metabolically oriented dietary strategies and micronutrient repletion. Ketogenic approaches are particularly relevant because β-hydroxybutyrate can sustain neuronal ATP production even when OXPHOS is impaired. Implementation should nonetheless be individualized, as multiple factors may influence both feasibility and response. For instance, some evidence suggests ketogenic interventions may be more consistently effective in men (Smolensky et al., 2023; Jiao et al., 2025; Zhang et al., 2025), potentially reflecting sex-specific differences in metabolic regulation. In females, metabolic demands fluctuate across the menstrual cycle, with the luteal phase often associated with increased carbohydrate cravings (Souza et al., 2018), which may reflect hormonally mediated shifts in energy utilization and substrate preference in presumptive embryos (Sussman et al., 2013; Souza et al., 2018).

Pronounced circadian instability - identified by actigraphy-confirmed rest-activity fragmentation, evening chronotype, or PER3/CLOCK risk genotypes - carries particular mechanistic rationale for rhythm-stabilizing approaches, including interpersonal and social rhythm therapy (IPSRT), structured sleep-wake scheduling, and light-based interventions designed to restore circadian alignment (Metwally El-Sayed et al., 2026). These approaches address the upstream circadian input to mitochondrial dysfunction proposed in this model and have the additional advantage of being combinable with pharmacotherapy without pharmacokinetic interaction.

Heightened inflammatory activity - indexed by elevated high-sensitivity CRP, IL-6, or TNF-α, points toward adjunctive strategies aimed at reducing immune activation, including dietary modification and supplementation with anti-inflammatory compounds such as omega-3 fatty acids (Eslahi et al., 2023; Lastretti et al., 2026). Much attention should nonetheless be paid to the quality of the formulation, as this substantially affects bioavailability and effect magnitude (Lastretti et al., 2026). This profile is also the one most likely to co-occur with gut-brain disruption, and microbiome-directed interventions - including probiotic formulations with demonstrated barrier-restoring properties and dietary patterns supporting SCFA-producing taxa - may act synergistically with anti-inflammatory approaches to restore intestinal integrity and reduce systemic signaling that contributes to central dysfunction (Kumar et al., 2026).

Where neuroendocrine dysregulation is prominent - particularly involving altered stress responsivity, cortisol signaling, elevated hair cortisol concentration, or a history of early-life trauma - stress-axis stabilization becomes a priority target. Psychotherapeutic interventions such as CBT, FFT, and Mindfulness-Based Stress Reduction (MBSR) may help reduce chronic HPA activation and improve regulatory stability (Milic et al., 2025), with mood-stabilizing effects likely mediated in part through downstream improvements in mitochondrial metabolic efficiency and inflammatory tone, consistent with the cascade proposed in this model.

Critically, these biological profiles are not mutually exclusive. The model predicts that the greatest illness severity and poorest treatment response will occur where dysregulation is co-occurring across multiple nodes - particularly the combination of mitochondrial insufficiency, elevated inflammation, and circadian instability. For this presentation, combined or sequential interventions targeting multiple domains simultaneously are likely required to achieve sustained mood stabilization. This framework therefore supports a shift toward selecting treatments based on their functional impact on dysregulated biological systems, and the primary research priority it implies is the development and validation of multimodal biological profiling tools capable of identifying which nodes are most dysregulated in a given patient, and the design of stratified trials testing whether matching intervention to biological profile improves outcomes beyond standard care.

## Conclusion

BD is a multifactorial and complex mental disorder with a diverse set of symptoms, different mood states, cognitive disturbances, and various courses of illness. The pathophysiology of BD is difficult to determine due to its complexity. However, it is clear that there is an overlap of several factors, including genetic predisposition, neurochemical imbalance, metabolic dysfunction, immune system activation, and exposure to stressors. The complexity and heterogeneity of BD are the main obstacles to the development of a standard treatment for this disorder. The main reasons for the disorder include the disruption of top-down control from the prefrontal cortices, leading to an imbalance in activity between cortical and subcortical, especially limbic structures. Another area of interest in BD is mitochondrial dysfunction, energy metabolism, oxidative stress, immune dysregulation, and altered microbiota-gut-brain axis. In this regard, it is important to note that BD is a systemic disorder. Further studies of BD require the development of a unified integrative mechanism-based model that could account for the genetic, molecular, brain circuit, and behavior levels of this disorder. As well as require to consider individual differences of a biological marker, trauma history, metabolic and inflammatory status, etc. This approach can target different pathways that may be the mechanism of several symptoms at the same time and increase treatment efficacy and long-term remission.
